# Application of Machine Learning for Patients With Cardiac Arrest: Systematic Review and Meta-Analysis

**DOI:** 10.2196/67871

**Published:** 2025-03-10

**Authors:** Shengfeng Wei, Xiangjian Guo, Shilin He, Chunhua Zhang, Zhizhuan Chen, Jianmei Chen, Yanmei Huang, Fan Zhang, Qiangqiang Liu

**Affiliations:** 1 Department of Emergency Medicine The First Affiliated Hospital, Sun Yat-sen University Guangzhou China

**Keywords:** cardiac arrest, machine learning, prognosis, systematic review, artificial intelligence, AI

## Abstract

**Background:**

Currently, there is a lack of effective early assessment tools for predicting the onset and development of cardiac arrest (CA). With the increasing attention of clinical researchers on machine learning (ML), some researchers have developed ML models for predicting the occurrence and prognosis of CA, with certain models appearing to outperform traditional scoring tools. However, these models still lack systematic evidence to substantiate their efficacy.

**Objective:**

This systematic review and meta-analysis was conducted to evaluate the prediction value of ML in CA for occurrence, good neurological prognosis, mortality, and the return of spontaneous circulation (ROSC), thereby providing evidence-based support for the development and refinement of applicable clinical tools.

**Methods:**

PubMed, Embase, the Cochrane Library, and Web of Science were systematically searched from their establishment until May 17, 2024. The risk of bias in all prediction models was assessed using the Prediction Model Risk of Bias Assessment Tool.

**Results:**

In total, 93 studies were selected, encompassing 5,729,721 in-hospital and out-of-hospital patients. The meta-analysis revealed that, for predicting CA, the pooled C-index, sensitivity, and specificity derived from the imbalanced validation dataset were 0.90 (95% CI 0.87-0.93), 0.83 (95% CI 0.79-0.87), and 0.93 (95% CI 0.88-0.96), respectively. On the basis of the balanced validation dataset, the pooled C-index, sensitivity, and specificity were 0.88 (95% CI 0.86-0.90), 0.72 (95% CI 0.49-0.95), and 0.79 (95% CI 0.68-0.91), respectively. For predicting the good cerebral performance category score 1 to 2, the pooled C-index, sensitivity, and specificity based on the validation dataset were 0.86 (95% CI 0.85-0.87), 0.72 (95% CI 0.61-0.81), and 0.79 (95% CI 0.66-0.88), respectively. For predicting CA mortality, the pooled C-index, sensitivity, and specificity based on the validation dataset were 0.85 (95% CI 0.82-0.87), 0.83 (95% CI 0.79-0.87), and 0.79 (95% CI 0.74-0.83), respectively. For predicting ROSC, the pooled C-index, sensitivity, and specificity based on the validation dataset were 0.77 (95% CI 0.74-0.80), 0.53 (95% CI 0.31-0.74), and 0.88 (95% CI 0.71-0.96), respectively. In predicting CA, the most significant modeling variables were respiratory rate, blood pressure, age, and temperature. In predicting a good cerebral performance category score 1 to 2, the most significant modeling variables in the in-hospital CA group were rhythm (shockable or nonshockable), age, medication use, and gender; the most significant modeling variables in the out-of-hospital CA group were age, rhythm (shockable or nonshockable), medication use, and ROSC.

**Conclusions:**

ML represents a currently promising approach for predicting the occurrence and outcomes of CA. Therefore, in future research on CA, we may attempt to systematically update traditional scoring tools based on the superior performance of ML in specific outcomes, achieving artificial intelligence–driven enhancements.

**Trial Registration:**

PROSPERO International Prospective Register of Systematic Reviews CRD42024518949; https://www.crd.york.ac.uk/prospero/display_record.php?RecordID=518949

## Introduction

### Background

Cardiac arrest (CA) remains a critical challenge in contemporary medicine, characterized by a dismally low survival rate and poor prognosis, and, therefore, has garnered global attention [1]. CA can be classified by the occurrence location into in-hospital CA (IHCA) and out-of-hospital CA (OHCA). Despite advancements in cardiopulmonary resuscitation techniques, global registry data indicate that the incidence and survival rates of CA have not significantly improved. The incidence of IHCA in the United States increased to 900 to 1000 per 100,000 hospitalized patients between 2008 and 2017, compared to 160 per 100,000 in the United Kingdom from 2011 to 2013 and 840 per 100,000 in China as of 2020 [2-4]. Meanwhile, the estimated averages of incidence of OHCA under emergency medical services (EMS) in North America, Asia, and Europe from 2010 to 2020 were 47.3, 45.9, and 40.6 per 100,000 people, respectively. The estimated averages of the survival rates of IHCA from 2010 to 2020 were 25% in the United States, 18% in the United Kingdom, and only 9.4% in China. For OHCA, the estimated averages of the survival rates during this same period were 10% to 12% in the United States, 8% in Europe, and just 3.6% in Asia [2,5]. These low survival rates also impose significant economic burdens on nations. According to relevant reviews, the cost-effectiveness threshold for CA ranged from US $20,000 to US $150,000 per quality-adjusted life year, with each life saved potentially reducing costs by US $19,000 to US $71,000 per case [6].

Although efforts to establish CA centers independently began in various regions of the United States as early as 2000 to 2010 [7] and Germany initiated CA center certification throughout the country [8] in August 2019 aiming to provide evidence-based, bundled care to improve CA survival rates, CA remains a formidable clinical challenge. If resuscitation is not timely, the patient may lose consciousness within approximately 10 seconds, with irreversible hypoxic-ischemic brain injury occurring within 4 minutes [9], and if resuscitation is delayed beyond 10 minutes, survival is practically impossible [10,11]. Thus, early prediction and identification of CA are critical factors in preventing death and poor outcomes and represent a major challenge that requires urgent clinical attention.

### Objectives

However, there is a scarcity of efficient, internationally recognized, and universally accepted assessment tools for early prediction and identification of CA risk and adverse outcomes. In recent years, with the rapid advancement of artificial intelligence (AI), many researchers have used machine learning (ML) to address clinical challenges. Commonly used ML approaches can be broadly categorized into supervised and unsupervised learning. In the context of supervised ML, clinical predictors can be incorporated into various models. In these models, their parameters are adjusted based on outcome variables to generate predictions regarding the probability of positive event occurrence [12]. It is now common to see ML being used to predict disease progression and even to diagnose and treat complex diseases effectively. For instance, in 2019, several authors, including Hatib et al [13] and Wijnberge et al [14], successfully predicted intraoperative hypotensive events using ML, leading to the clinical translation of these models into products that significantly enhanced patient safety during surgical anesthesia [15]. By 2023, some researchers had similarly affirmed the substantial potential of ML models in cancer detection, prognosis, and treatment, recognizing their exciting discoveries and contributions to advancing medical practice [16]. The aforementioned studies were based on supervised ML and the extensive use of interpretable clinical features to construct predictive models and simultaneously demonstrate the promising predictive performance of ML in clinical events across various fields. In this context, some researchers have also developed different ML models for risk prediction in CA. Recent reviews by Sem et al [17] and Chen et al [18] indicate that ML appears to exhibit high accuracy in both the management and risk prediction of CA. However, these reviews do not quantitatively synthesize the results of ML models, which significantly limits our ability to interpret the specific value of various ML models in CA applications and the selection of appropriate models. Therefore, we conducted this systematic review and meta-analysis to review the predictive performance of ML for the occurrence of CA, good neurological prognosis after CA, mortality, and the return of spontaneous circulation (ROSC) after CA to provide evidence-based guidance for the development and updating of simple prediction tools with high accuracy and direct access to results.

## Methods

### Study Registration

This study was conducted in adherence to the PRISMA (Preferred Reporting Items for Systematic Reviews and Meta-Analyses) guidelines and prospectively registered with PROSPERO (International Prospective Register of Systematic Reviews; ID CRD42024518949). The detailed PRISMA checklist is presented in Multimedia Appendix 1.

### Eligibility Criteria

Detailed inclusion and exclusion criteria were defined to screen the original studies relevant to our systematic review from the retrieved literature (Textbox 1).

**Inclusion and exclusion criteria for original studies.**

**Inclusion criteria**

- Study type: the included studies must be case-control, cohort, nested case-control, case-cohort, or cross-sectional studies.
- Model construction: although some studies, due to limited sample sizes, lacked independent external validation, we could not dismiss their contributions. In our analysis, it was necessary to synthesize results from the training and validation sets to assess the presence of severe overfitting. Therefore, those with no external validation were also included.
- Outcomes: studies that comprehensively constructed machine learning (ML) models for cardiac arrest (CA) occurrence prediction or clinical outcomes following CA were selected.
- Language: we included original studies in English.

**Exclusion criteria**

- Study type: studies categorized as meta-analyses, reviews, guidelines, expert opinions, or conference abstracts and not fully peer reviewed and published were removed.
- Model construction: studies with only risk factor analysis but no construction of a complete ML model were excluded, those with a limited number of samples (<20) were not included, and those only focusing on the accuracy of univariate predictors were removed.
- Outcomes: in existing ML studies, model performance was assessed using the receiver operating characteristic curve, C-statistic, sensitivity, specificity, accuracy, recall, precision, confusion matrix, or *F_1_*-score. However, a few original studies that lacked at least one of these metrics and, therefore, did not evaluate model performance adequately were excluded.
- Language: non–English-language original studies were excluded.

### Data Sources and Search Strategy

A systematic search of the PubMed, Embase, Cochrane Library, and Web of Science databases was carried out from their inception to May 17, 2024. The search strategy involved controlled vocabulary and free-text terms, with no restrictions on geographical location or publication year. The detailed search strategy is presented in Multimedia Appendices 2-5.

### Study Selection and Data Extraction

The retrieved studies were imported into EndNote X9 (Clarivate Analytics). Their titles and abstracts were reviewed. After the exclusion of duplicates, the preliminary eligible original studies were selected and their full texts downloaded for determining the final inclusion. An electronic spreadsheet was prepared to extract the following information: first author, publication year, author’s country, study type, patient source, prediction events, data balance, location of CA occurrence, number of cases with study events, total number of cases, number of cases in the training and validation sets, method of validation set generation, missing data–handling methods, and types of models used. Study selection and data extraction were independently conducted by 2 researchers. Disagreements were discussed and resolved with a third author.

### Risk of Bias in the Studies

The Prediction Model Risk of Bias Assessment Tool (PROBAST) was used to assess the risk of bias in all the included original studies. PROBAST comprises several questions across 4 domains—participant, predictor, outcome, and statistical analysis—which reflect the overall risk of bias and applicability. These domains consist of 2, 3, 6, and 9 questions, respectively, each with 3 possible answers (*Yes* or *Probably* *yes*, *No* or *Probably no*, and *No information*). A domain was classified as high risk if any question was answered with *No* or *Probably no*. Conversely, a domain was regarded as low risk only if every question was answered with *Yes* or *Probably yes*. The overall risk of bias was assessed as low when all domains were deemed to be low risk. When at least one domain was high risk, the overall risk of bias was rated as high. In total, 2 authors independently assessed the risk of bias using PROBAST and cross-checked their findings. Any discrepancies were addressed by consulting with a third author to reach agreement.

### Outcomes

The primary outcome measure was the C-index, which reflects the predictive ability of ML models for IHCA and OHCA. However, we found that the C-index might not have accurately described the predictive performance of ML for positive events, particularly in models built on severely imbalanced data, as these original studies often suffered from such imbalance. This limitation was evident in predicting the occurrence of IHCA and OHCA, the good cerebral performance category score 1 to 2 (CPC 1-2), mortality, and ROSC. Therefore, in addition to the C-index, our primary outcome measures encompassed sensitivity and specificity. Our secondary outcome was the frequency of variables used in the ML models.

### Synthesis Methods

A meta-analysis of the C-index, a measure of the general accuracy of ML models, was carried out. When the 95% CI and SE for the C-index were missing in some studies, the SE was estimated based on the study by Debray et al [19]. Due to the differences in the included variables and inconsistent parameters among the ML models, random-effects models were prioritized in the meta-analysis of the C-index. In addition, a meta-analysis on sensitivity and specificity was conducted through a bivariate mixed-effects model based on diagnostic 2 × 2 tables. However, most original studies did not report these tables. In such cases, we used the following methods to calculate the 2 × 2 tables: (1) calculation based on sensitivity, specificity, precision, and case numbers; and (2) calculation based on the best Youden index to extract sensitivity and specificity, followed by case number integration. Nevertheless, this method allowed for meta-analysis only when there were ≥4 models. For <4 models, we presented the range of sensitivity and specificity. Our meta-analysis was conducted in R (version 4.2.0; R Foundation for Statistical Computing).

## Results

### Study Selection

**Figure 1:**
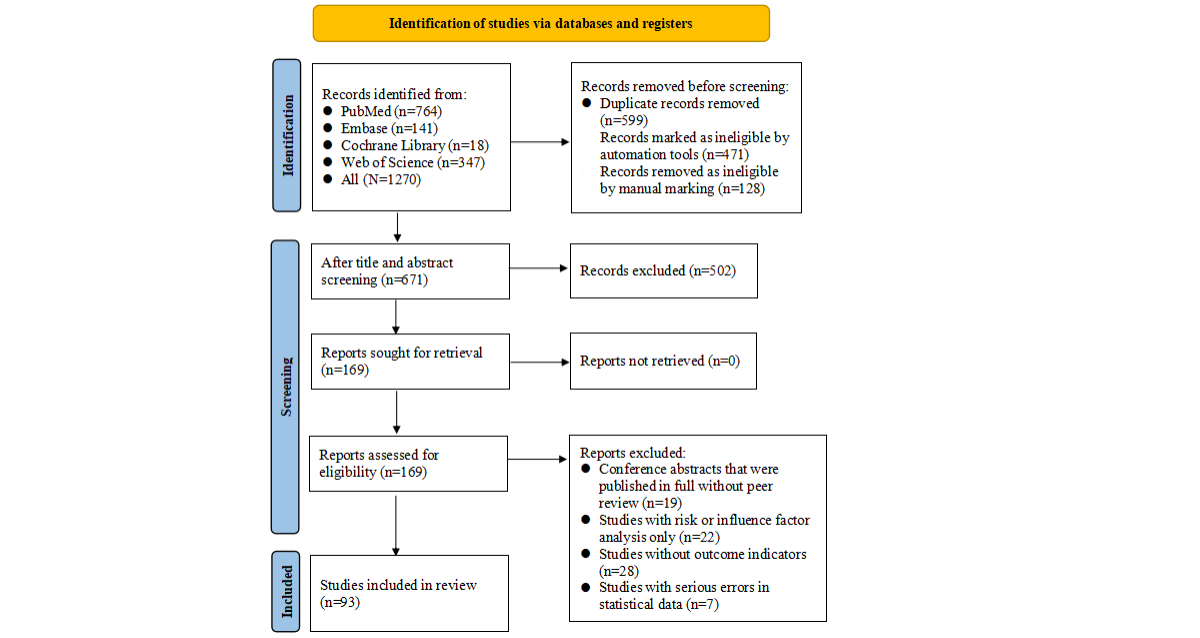
The PRISMA (Preferred Reporting Items for Systematic Reviews and Meta-Analyses) flow diagram for study selection.

A total of 1270 articles were obtained from databases, with 599 (47.17%) being duplicates. Among these 599 duplicates, 471 (78.6%) were found to be duplicates via software, and 128 (21.4%) were manually identified as duplicates. After the elimination of duplicates, 671 articles were screened by title and abstract, with 169 (25.2%) being selected for full-text review. After the exclusion of conference abstracts published in full text without peer review (19/169, 11.2%), studies with risk factor analyses but no complete ML models (22/169, 13%), studies lacking outcome indicators (28/169, 16.6%), and studies with severe statistical errors (7/169, 4.1%), a total of 93 articles were included finally. The detailed process is illustrated in Figure 1.

### Study Characteristics

The 93 selected studies were published between 2011 and 2024, covering 14 countries, primarily South Korea, China, Japan, the United States, and Singapore. Among the 93 studies, there were 23 (25%) prospective cohort studies and 3 (3%) case-control studies, with the remainder (67/93, 72%) being retrospective cohort studies. Data for 26% (24/93) of the studies were sourced from multiple centers, whereas 37% (34/93) of the studies used data from registry databases and the rest (35/93, 38%) were single-center studies. In 30% (28/93) of the studies, the predicted outcome was the occurrence of CA. In 42 studies, the predicted outcome was the neurological prognosis of patients with CA, with 10 (24%) studies focused on patients with IHCA and the remainder (n=32, 76%) focused on patients with OHCA. In 27% (25/93) of the studies, the predicted outcome was CA mortality, and in 12% (11/93) of the studies, the predicted outcome was ROSC in patients with CA. The 93 studies collectively encompassed a total of 5,729,721 cases, including 1,737,085 OHCA cases and 3,992,636 IHCA cases. Regarding the predictive models constructed, 81% (75/93) of the studies had independent validation sets, but only 27% (25/93) used external validation, primarily using k-fold cross-validation and random-sampling internal validation methods. A total of 34% (32/93) of the studies described methods to prevent data overfitting, mainly through cross-validation. In total, 17 types of ML models were involved, with logistic regression (LR), random forest (RF), deep learning, and decision trees (DTs) being the most prominent. In addition, these studies validated several previously established scoring tools, including the Cardiac Arrest Neurological Prognosis score, distance scoring system, Emergency Department In-Hospital Cardiac Arrest Score, FACTOR score, Modified Early Warning Score, National Early Warning Score, National Early Warning Score 2, OHCA score, proposed scoring system, Rapid Emergency Medicine Score, Simplified Acute Physiology Score II, Cardiac Arrest Hospital Prognosis score, and ROSC after CA score. The details of the included studies are shown in Tables 1 and 2.

**Table 1:** Characteristics of the included studies.

Study	Year of publication	Country of first author	Study type (case-control, cohort study [retrospective or prospective], nested cohort study, or case-cohort study)	Patient sources (single center, multicenter, or registration database)	Predictive events
Wang et al [20]	2024	Taiwan, China	Retrospective cohort study	Multicenter	Cardiac arrest
Raheem et al [21]	2024	Pakistan	Retrospective cohort study	Single center	Cardiac arrest
Amacher et al [22]	2024	Switzerland	Prospective cohort study	Single center	In-hospital mortality and CPC 3-5^a^
Cho et al [23]	2024	Republic of Korea	Retrospective cohort study	Single center	Cardiac arrest
Shin et al [24]	2024	Republic of Korea	Retrospective cohort study	Single center	Cardiac arrest
Ding et al [25]	2024	China	Prospective cohort study	Single center	CPC 1-2^b^ and in-hospital mortality
Nishioka et al [26]	2024	Japan	Retrospective cohort study	Multicenter	CPC 3-5
Kajino et al [27]	2024	Japan	Retrospective cohort study	Registration database	CPC 1-2
Pham et al [28]	2024	United States	Prospective cohort study	Multicenter	Cardiac arrest
Rahadian et al [29]	2024	Japan	Prospective cohort study	Registration database	VF^c^ or VT^d^
Wang et al [30]	2024	Taiwan, China	Retrospective cohort study	Registration database	Cardiac arrest
Tsai et al [31]	2024	Taiwan, China	Retrospective cohort study	Single center	CPC 1-2
Schweiger et al [32]	2024	Switzerland	Prospective cohort study	Single center	In-hospital mortality
Caputo et al [33]	2024	Switzerland	Prospective cohort study	Registration database	ROSC^e^
Lu et al [34]	2023	Taiwan, China	Retrospective cohort study	Single center	Cardiac arrest
Dünser et al [35]	2023	Austria	Retrospective cohort study	Single center	NROSC^f^ and CPC 3-5
Bang et al [36]	2023	Republic of Korea	Retrospective cohort study	Multicenter	In-hospital mortality
Zhang et al [37]	2023	China	Retrospective cohort study	Multicenter	In-hospital mortality
Li and Xing [38]	2023	China	Retrospective cohort study	Single center	CPC 3-5 and NROSC
Ding et al [39]	2023	China	Retrospective cohort study	Single center	Cardiac arrest
Uehara et al [40]	2023	Japan	Prospective cohort study	Multicenter	CPC 1-2
Shin et al [41]	2023	Republic of Korea	Retrospective cohort study	Registration database	CPC 1-2 and ROSC
Kawai et al [42]	2023	Japan	Retrospective cohort study	Single center	CPC 3-5
Imamura et al [43]	2023	Japan	Retrospective cohort study	Multicenter	30-day mortality
Hessulf et al [44]	2023	Sweden	Retrospective cohort study	Multicenter	30-day survival
Yoon et al [45]	2023	Republic of Korea	Retrospective cohort study	Single center	CPC 3-5
Chang et al [46]	2023	Republic of Korea	Retrospective cohort study	Registration database	ROSC, survival to discharge, and CPC 1-2
Wang et al [47]	2023	China	Retrospective cohort study	Multicenter	ROSC
Shinada et al [48]	2023	Japan	Retrospective cohort study	Registration database	CPC 1-2
Xu et al [49]	2022	China	Case-control study	Single center	Cardiac arrest
Tsai et al [50]	2022	Taiwan, China	Retrospective cohort study	Registration database	Cardiac arrest
Tang et al [51]	2022	China	Retrospective cohort study	Registration database	Cardiac arrest
Kim et al [52]	2022	Republic of Korea	Retrospective cohort study	Registration database	Cardiac arrest
Chae et al [53]	2022	Republic of Korea	Retrospective cohort study	Single center	Cardiac arrest
Sun et al [54]	2022	Taiwan, China	Retrospective cohort study	Single center	Cardiac arrest
Wong et al [55]	2022	Singapore	Prospective cohort study	Multicenter	Survival to discharge
Tran et al [56]	2022	United States	Prospective cohort study	Registration database	In-hospital mortality
Rajendram et al [57]	2022	Singapore	Retrospective cohort study	Multicenter	Survival to discharge and CPC 1-2
Rafi et al [58]	2022	France	Retrospective cohort study	Single center	Cardiac arrest
Liu et al [59]	2022	Singapore	Retrospective cohort study	Registration database	ROSC
Lin et al [60]	2022	Taiwan, China	Retrospective cohort study	Registration database	CPC 1-2
Kawai et al [61]	2022	Japan	Retrospective cohort study	Multicenter	CPC 1-2
Itagaki et al [62]	2022	Japan	Retrospective cohort study	Single center	Brain death
Harris et al [63]	2022	United States	Retrospective cohort study	Registration database	Prehospital ROSC in pediatric OHCA^g^
Harford et al [64]	2022	United States	Retrospective cohort study	Registration database	CPC 1-2
Harford et al [65]	2022	United States	Retrospective cohort study	Registration database	CPC 1-2
Chung et al [66]	2021	Taiwan, China	Retrospective cohort study	Single center	CPC 1-2
Chi et al [67]	2021	Taiwan, China	Retrospective cohort study	Registration database	In-hospital mortality
Wang et al [68]	2021	China	Retrospective cohort study	Multicenter	CPC 1-2
Bae et al [69]	2021	Republic of Korea	Retrospective cohort study	Single center	CPC 3-5
Mueller et al [70]	2021	Austria	Prospective cohort study	Single center	CPC 1-2
Lee et al [71]	2021	Republic of Korea	Retrospective cohort study	Multicenter	Cardiac arrest
Lim et al [72]	2021	Republic of Korea	Prospective cohort study	Registration database	CPC 1-2
Lo and Siu [73]	2021	Hong Kong, China	Retrospective cohort study	Registration database	ROSC
Lonsain et al [74]	2021	Belgium	Retrospective cohort study	Single center	24-hour survival
Nishioka et al [75]	2021	Japan	Prospective cohort study	Registration database	CPC 1-2
Beom et al [76]	2021	Republic of Korea	Prospective cohort study	Multicenter	Survival to discharge and CPC 1-2
Cheng et al [77]	2021	Taiwan, China	Retrospective cohort study	Single center	CPC 1-2
Kim et al [78]	2021	Republic of Korea	Retrospective cohort study	Registration database	Survival to discharge and CPC 1-2
Seo et al [79]	2021	Republic of Korea	Prospective cohort study	Registration database	CPC 1-2
Song et al [80]	2021	Republic of Korea	Retrospective cohort study	Single center	CPC 3-5
Sun et al [81]	2021	Hong Kong, China	Retrospective cohort study	Multicenter	ROSC
Youn et al [82]	2021	Republic of Korea	Prospective cohort study	Multicenter	Significant coronary artery disease among survivors of OHCA without STE^h^
Heo et al [83]	2021	Republic of Korea	Prospective cohort study	Multicenter	CPC 3-5
Wang et al [84]	2020	China	Retrospective cohort study	Registration database	CPC 1-2
Hong et al [85]	2020	Republic of Korea	Retrospective cohort study	Single center	Cardiac arrest
Cho et al [86]	2020	Republic of Korea	Retrospective cohort study	Single center	Cardiac arrest
Hirano et al [87]	2020	Japan	Retrospective cohort study	Registration database	Death at 1 month or survival with poor neurological function (CPC 3-5) and 30-day mortality
Okada et al [88]	2020	Japan	Prospective cohort study	Registration database	CPC 1-2
Liu et al [89]	2020	Singapore	Retrospective cohort study	Registration database	ROSC
Elola et al [90]	2020	Spain	Retrospective cohort study	Registration database	Cardiac arrest
Hsieh et al [91]	2020	Taiwan, China	Retrospective cohort study	Registration database	Cardiac arrest
Baldi et al [92]	2020	Italy	Prospective cohort study	Multicenter	Survival to hospital admission
Li et al [93]	2019	China	Case-control study	Multicenter	Cardiac arrest
Srivilaithon et al [94]	2019	Thailand	Case-control study	Single center	Cardiac arrest
Lee et al [95]	2019	Republic of Korea	Retrospective cohort study	Single center	CPC 3-5
Liu et al [96]	2019	Taiwan, China	Retrospective cohort study	Single center	Cardiac arrest
Jang et al [97]	2019	Republic of Korea	Retrospective cohort study	Single center	Cardiac arrest
Seki et al [98]	2019	Japan	Prospective cohort study	Multicenter	1-year survival
Park et al [99]	2019	Republic of Korea	Retrospective cohort study	Registration database	CPC 1-2
Kwon et al [100]	2019	Republic of Korea	Retrospective cohort study	Registration database	CPC 1-2 and survival to discharge
Kong et al [101]	2019	Republic of Korea	Prospective cohort study	Multicenter	CPC 1-2 and survival to discharge
Harford et al [102]	2019	United States	Retrospective cohort study	Registration database	CPC 1-2
Kwon et al [103]	2018	Republic of Korea	Retrospective cohort study	Multicenter	Cardiac arrest and in-hospital mortality
Chang et al [104]	2018	Taiwan, China	Retrospective cohort study	Single center	Cardiac arrest
Shin et al [105]	2018	Republic of Korea	Retrospective cohort study	Multicenter	CPC 1-2
Lee et al [106]	2017	Republic of Korea	Retrospective cohort study	Single center	Survival to hospital discharge
Liu et al [107]	2015	Singapore	Retrospective cohort study	Single center	Cardiac arrest
Goto et al [108]	2014	Japan	Retrospective cohort study	Registration database	CPC 1-2 and 30-day survival
Goto et al [109]	2013	Japan	Retrospective cohort study	Registration database	CPC 1-2 and 30-day survival
Hock Ong et al [110]	2012	Singapore	Prospective cohort study	Single center	Cardiac arrest and in-hospital mortality
Hayakawa et al [111]	2011	Japan	Prospective cohort study	Registration database	CPC 1-2

^a^CPC 3-5: poor cerebral performance category score 3 to 5.

^b^CPC 1-2: good cerebral performance category score 1 to 2.

^c^VF: ventricular fibrillation.

^d^VT: ventricular tachycardia.

^e^ROSC: return of spontaneous circulation.

^f^NROSC: non-ROSC.

^g^OHCA: out-of-hospital cardiac arrest.

^h^STE: ST segment elevation.

**Table 2:** Analytical characteristics of the included studies.

Study	Balance of data (balanced or unbalanced)	Location of CA^a^	Number of cases of studied events	Total number of cases	Number of cases in the training set	Generation of validation set	Number of cases in the validation set	Handling method for missing values	Model type
Wang et al [20]	Unbalanced	In hospital	474	224,413	182,716	External validation	41,697	Deletion	Logistic regression, National Early Warning Score, and Modified Early Warning Score
Raheem et al [21]	Unbalanced	In hospital	5483	97,353	77,886	Internal validation	19,467	Deletion	Artificial neural network, random forest, and logistic regression
Amacher et al [22]	Balanced	In hospital and out of hospital	IHM^b^: 309; CPC 3-5^c^: 309	713	—^d^	—	713	No processing	Out-of-hospital CA score, the Cardiac Arrest Hospital Prognosis score, and logistic regression
Cho et al [23]	Unbalanced	In hospital	228	95,607	—	External validation	95,607	Deletion	Deep learning, Modified Early Warning Score, and National Early Warning Score
Shin et al [24]	Unbalanced	In hospital	198	1995	970	External validation	1025	Deletion	Deep learning, logistic regression, random forest, and National Early Warning Score
Ding et al [25]	Balanced	In hospital	CPC 1-2^e^: 20; IHM: 30	53	—	Internal validation	53	Deletion	Logistic regression and Cox regression
Nishioka et al [26]	Balanced	Out of hospital	6486	7587	3337	External validation	4250	Supplement	Logistic regression
Kajino et al [27]	Unbalanced	Out of hospital	11,411	302,799	149,425	Internal validation	153,374	Deletion	Deep learning
Pham et al [28]	Balanced	Out of hospital	210	434	231	External validation (multicenter)	203	—	Logistic regression
Rahadian et al [29]	Unbalanced	Out of hospital	860	20,713	17,162	—	3551	Imputation	Logistic regression, LASSO^f^, and random forest
Wang et al [30]	Unbalanced	Out of hospital	84	48,371	32,244	—	16,127	—	Logistic regression
Tsai et al [31]	Balanced	Out of hospital	CPC 1-2: 127	443	265	Internal validation	178	—	Logistic regression
Schweiger et al [32]	Balanced	Out of hospital	120	291	138	Internal validation	153	Supplement	FACTOR score
Caputo et al [33]	Unbalanced	Out of hospital	2719	12,577	—	Internal validation	12,577	—	Logistic regression
Lu et al [34]	Unbalanced	Emergency department	636	316,465	237,349	Random sampling	79,116	Supplement	Logistic regression, random forest, National Early Warning Score 2, and XGBoost^g^
Dünser et al [35]	Balanced	Operating rooms and departments outside the ICU^h^	NROSC^i^: 390; CPC 3-5: 559	630	—	Internal validation	630	No processing	Random forest
Bang et al [36]	Balanced	In hospital	411	1133	754	Random sampling	379	Deletion	Logistic regression
Zhang et al [37]	Balanced	In hospital	495	561	561	—	—	Deletion	Logistic regression
Li and Xing [38]	Balanced	In hospital	NROSC: 564; CPC 3-5: 229	851	851	Internal validation (bootstrap)	—	Deletion	Logistic regression
Ding et al [39]	Balanced	In hospital	1769	3592	2873	Internal validation	719	Deletion	Support vector machine, random forest, XGBoost, decision tree, and logistic regression
Uehara et al [40]	Unbalanced	Out of hospital	71	8422	4239	Random sampling (1:1)	4183	Deletion	Logistic regression
Shin et al [41]	Unbalanced	Out of hospital	ROSC^j^: 3095; CPC 1-2: 990	16,992	—	Random sampling	—	Deletion	K-nearest neighbor, decision tree, random forest, support vector machine, logistic regression, and deep learning
Kawai et al [42]	Balanced	Out of hospital	254	321	257	Random sampling (8:2）	64	Deletion	Deep learning
Imamura et al [43]	Balanced	Out of hospital	172	274	194	External validation (multicenter)	80	Deletion	Logistic regression
Hessulf et al [44]	Unbalanced	Out of hospital	6191	55,615	44,492	Random sampling	11,123	Algorithm	XGBoost
Yoon et al [45]	Balanced	Out of hospital	74	131	—	External validation	131	Deletion	Logistic regression
Chang et al [46]	Unbalanced	Out of hospital	ROSC: 11,996; STD^k^: 11,833; 30-day survival: 7760; CPC 1-2: 3673	157,654	157,654	Internal validation	—	Deletion	LightGBM^l^
Wang et al [47]	Unbalanced	Out of hospital	156	2685	2685	Internal validation	—	Deletion	Logistic regression
Shinada et al [48]	Unbalanced	Out of hospital	1128	5340	4286	Internal validation	1054	Deletion	Naïve Bayes
Xu et al [49]	Balanced	Emergency department and out of hospital	150	600	600	—	—	Deletion	Logistic regression
Tsai et al [50]	Unbalanced	Emergency department	623	325,502	325,502	—	—	No processing	Logistic regression, Modified Early Warning Score, and National Early Warning Score
Tang et al [51]	Balanced	ICU	107	486	—	Internal validation	486	Algorithm	National Early Warning Score, random forest, artificial neural network, and deep learning
Kim et al [52]	Unbalanced	Emergency department	5431	1,350,693	1,080,554	Random sampling	270,139	Deletion	Logistic regression, XGBoost, artificial neural network, and logistic regression
Chae et al [53]	Unbalanced	In hospital	573	34,452	—	Random sampling	34,452	Supplement	Decision tree, random forest, logistic regression, and artificial neural network
Sun et al [54]	Unbalanced	Emergency department	240	145,557	—	External validation	145,557	Deletion	Emergency department, in-hospital CA score, Modified Early Warning Score, and Rapid Emergency Medicine Score
Wong et al [55]	Unbalanced	Out of hospital	855	4776	3582	Random sampling	1194	Deletion	Random forest
Tran et al [56]	Balanced	Out of hospital	996	2999	2999	Internal validation	—	Deletion	Logistic regression
Rajendram et al [57]	Unbalanced	Out of hospital	STD: 3549; CPC 1-2: 1754	24,897	—	External validation	24,897	Deletion	Random forest
Rafi et al [58]	Balanced	Out of hospital	410	820	—	Internal validation	820	Supplement (algorithm)	Logistic regression, random forest, and artificial neural network
Liu et al [59]	Unbalanced	Out of hospital	12,729	153,611	119,477	External validation (multicenter)	34,134	Deletion	Random forest
Lin et al [60]	Unbalanced	Out of hospital	160	3520	2816	Random sampling (8:2)	704	Deletion	Decision tree and random forest
Kawai et al [61]	Unbalanced	Out of hospital	286	8274	6620	Internal validation (cross-validation)	1654	Deletion	Artificial neural network
Itagaki et al [62]	Balanced	Out of hospital	BD^m^: 77	419	—	Internal validation (bootstrap)	419	Deletion	Logistic regression
Harris et al [63]	Unbalanced	Out of hospital	399	1726	1381	Random sampling	345	Supplement (algorithm)	Logistic regression, random forest, and LightGBM
Harford et al [64]	Unbalanced	Out of hospital	379	1798	957	Internal validation (cross-validation)	241 and 600	Deletion	LightGBM, XGBoost, decision tree, random forest, k-nearest neighbor, logistic regression, and deep learning
Harford et al [65]	Unbalanced	Out of hospital	670	9595	5750	Random sampling	1445 and 2400	Deletion	Deep learning
Chung et al [66]	Unbalanced	In hospital	94	796	637	Random sampling	159	No processing	Artificial neural network
Chi et al [67]	Balanced	In hospital	87,311	168,693	168,693	—	—	Deletion	HVec^n^
Wang et al [68]	Unbalanced	In hospital	46	159	80	External validation (multicenter)	79	Deletion	CANP^o^ score
Bae et al [69]	Balanced	In hospital	643	982	671	External validation (prospective)	311	Deletion	Logistic regression
Mueller et al [70]	Balanced	In hospital	223	475	475	—	—	Deletion	Logistic regression
Lee et al [71]	Unbalanced	In hospital	425	332,371	173,368	External validation (multicenter)	159,003	Algorithm	Deep learning and Modified Early Warning Score
Lim et al [72]	Unbalanced	—	892	8240	4712	External validation (prospective)	3528	Deletion	Logistic regression
Lo and Siu [73]	Unbalanced	Out of hospital	2787	8157	6525	Random sampling	1632	Deletion	Logistic regression, random forest, and artificial neural network
Lonsain et al [74]	Balanced	Out of hospital	168	192	192	Internal validation	—	—	Logistic regression
Mueller et al [70]	Balanced	Out of hospital	761	1874	—	Internal validation	1874	—	Logistic regression
Nishioka et al [75]	Balanced	Out of hospital	382	2354	1329	External validation (prospective)	1025	Supplement (algorithm)	Logistic regression
Beom et al [76]	Balanced	Out of hospital	STD: 475; CPC 1-2: 315	1432	496 (survival prognosis validation group) and 489 (neurological prognosis validation group)	Random sampling (7:3)	STD: 227; CPC 1-2: 220	Deletion	Logistic regression
Cheng et al [77]	Unbalanced	Out of hospital	86	1071	—	Random sampling (9:1)	1071	Deletion	Logistic regression, XGBoost, and support vector machine
Kim et al [78]	Unbalanced	Out of hospital	1986	39,602	39,602	Internal validation	—	Deletion	Random forest, LightGBM, and artificial neural network
Seo et al [79]	Balanced	Out of hospital	105	5739	5739	Internal validation	—	Supplement (algorithm)	Random forest, XGBoost, and logistic regression
Song et al [80]	Balanced	Out of hospital	61	106	—	External validation	106	Deletion	Out-of-hospital CA score
Sun et al [81]	Unbalanced	Out of hospital	148	447	447	Internal validation	—	Deletion	Logistic regression
Youn et al [82]	Unbalanced	Out of hospital	127	331	—	Internal validation	331	Deletion	Random forest, CatBoost, and logistic regression
Heo et al [83]	Balanced	Out of hospital	704	903	631	External validation (prospective)	158 and 114	Mean	Ensemble learning and logistic regression
Wang et al [84]	Balanced	In hospital and out of hospital	114	262	262	Internal validation	—	No processing	Logistic regression
Hong et al [85]	Unbalanced	Emergency department	993	214,307	168,488	Random sampling	45,819	Supplement	Modified Early Warning Score, logistic regression, artificial neural network, and random forest
Cho et al [86]	Unbalanced	Inpatient ward	11	8039	—	External validation	8039	No processing	Modified Early Warning Score and deep learning
Hirano et al [87]	Balanced	Out of hospital	30-day mortality: 13,329	30,049	23,668	Internal validation (10-fold cross-validation)	6381	Deletion	Logistic regression, support vector machine, random forest, artificial neural network, and multilayer perceptron
Okada et al [88]	Unbalanced	Out of hospital	114	916	458	Internal validation	458	—	Logistic regression
Liu et al [89]	Unbalanced	Out of hospital	5190	63,059	44,141	Internal validation	18,918	—	ROSC after CA score and random forest
Elola et al [90]	Unbalanced	Out of hospital	55	162	96	Internal validation (5-fold cross-validation)	66	—	Random forest
Hsieh et al [91]	Unbalanced	Out of hospital	660	252,771	168,522	Internal validation	84,249	Deletion	Logistic regression
Baldi et al [92]	Unbalanced	Out of hospital	625	2709	1962	Internal validation	747	—	Logistic regression
Li et al [93]	Unbalanced	Emergency department	164	656	656	Random sampling	—	Supplement (algorithm)	Decision tree
Srivilaithon et al [94]	Unbalanced	Emergency department	250	1250	—	External validation	1250	Deletion	National Early Warning Score
Lee et al [95]	Balanced	In hospital	367	580	580	—	—	Deletion	Logistic regression
Liu et al [96]	Unbalanced	Emergency department	124	43,569	43,569	Internal validation	—	No processing	AdaBoost^p^, random forest, naïve Bayes, decision tree, logistic regression, artificial neural network, and deep learning
Jang et al [97]	Unbalanced	Emergency department	1568	523,852	261,926	—	261,926	Deletion	Artificial neural network, Modified Early Warning Score, logistic regression, and random forest
Seki et al [98]	Unbalanced	Out of hospital	432	7326	5718	External validation (prospective)	1608	Imputation	Random forest
Park et al [99]	Unbalanced	Out of hospital	2805	19,832	15,860	Random sampling (8:2)	3972	Deletion	Logistic regression, XGBoost, support vector machine, random forest, and artificial neural network
Kwon et al [100]	Unbalanced	Out of hospital	CPC 1-2: 3812; STD: 6435	36,190	28,045	—	8145	—	Deep learning, logistic regression, random forest, and support vector machine
Kong et al [101]	Unbalanced	Out of hospital	CPC 1-2: 156; STD: 251	737	524	External validation	213	—	Logistic regression
Harford et al [102]	Unbalanced	Out of hospital	250	2244	1584	Internal validation	660	Supplement (algorithm)	Deep learning
Kwon et al [103]	Unbalanced	In hospital	CA: 415; IHM: 795	50,359	46,725	External validation (multicenter)	3634	Supplement (median)	Deep learning, Modified Early Warning Score, logistic regression, and random forest
Chang et al [104]	Unbalanced	Emergency department	124	43,569	—	Internal validation	43,569	Supplement (mean)	Logistic regression, decision tree, random forest, and XGBoost
Shin et al [105]	Unbalanced	Out of hospital	CPC 1-2: 86	456	228	Internal validation	228	Deletion	Decision tree
Lee et al [106]	Unbalanced	Emergency department	21	111	—	Internal validation (bootstrap)	111	Deletion	Logistic regression and Simplified Acute Physiology Score II
Liu et al [107]	Unbalanced	Emergency department	52	1025	—	Internal validation (cross-validation)	1025	Deletion	Proposed scoring system and distance scoring system
Goto et al [108]	Unbalanced	Out of hospital	CPC 1-2: 205; 30-day survival: 581	5379	3693	External validation (prospective)	1686	Deletion	Decision tree
Goto et al [109]	Unbalanced	Out of hospital	CPC 1-2: 7769; 30-day survival: 16,332	390,226	307,896	Internal validation	82,330	Deletion	Decision tree
Hock Ong et al [110]	Unbalanced	Emergency department	CA: 43; IHM: 86	925	—	External validation	925	Deletion	Modified Early Warning Score and support vector machine
Hayakawa et al [111]	Unbalanced	Out of hospital	244	1497	862	External validation (prospective)	635	Deletion	Logistic regression

^a^CA: cardiac arrest.

^b^IHM: in-hospital mortality.

^c^CPC 3-5: poor cerebral performance category score 3 to 5.

^d^Not provided.

^e^CPC 1-2: good cerebral performance category score 1 to 2.

^f^LASSO: least absolute shrinkage and selection operator.

^g^XGBoost: Extreme Gradient Boosting.

^h^ICU: intensive care unit.

^i^NROSC: nonreturn of spontaneous circulation.

^j^ROSC: return of spontaneous circulation.

^k^STD: survival to discharge.

^l^LightGBM: Light Gradient-Boosting Machine.

^m^BD: brain death.

^n^HVec: hierarchical vectorizer.

^o^CANP: Cardiac Arrest Neurological Prognosis.

^p^AdaBoost: Adaptive Boosting.

### Risk of Bias in the Studies

**Figure 2:**
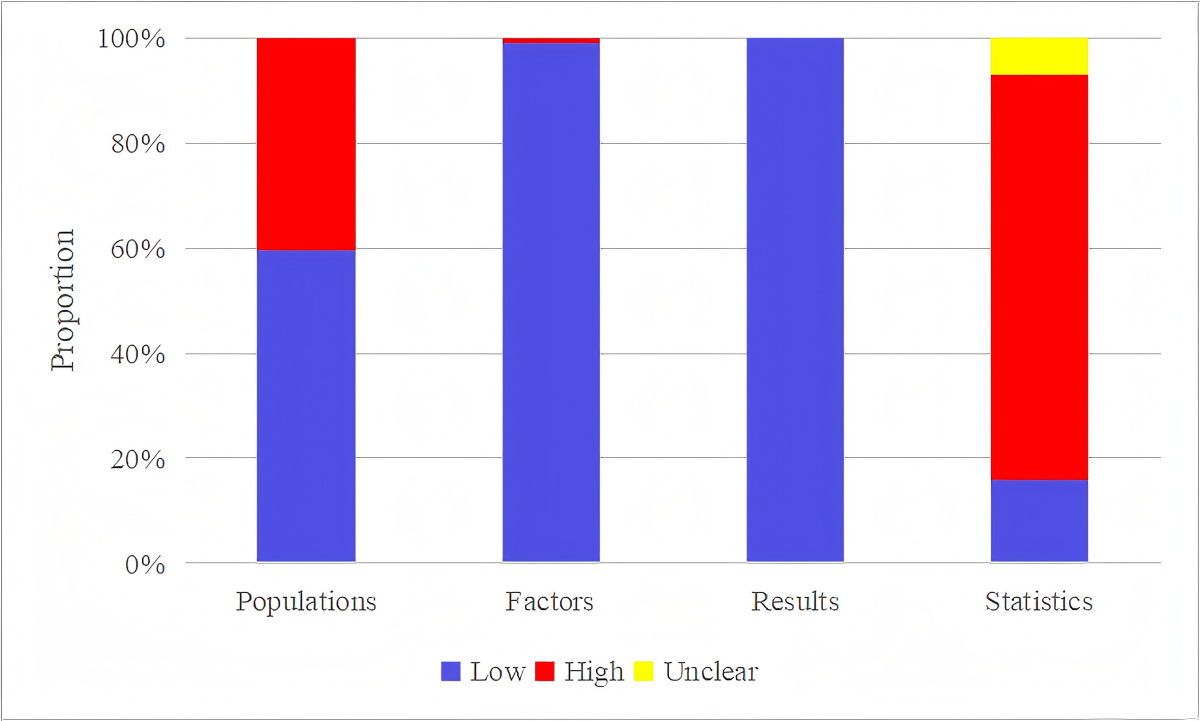
Assessment results for the risk of bias in the included models.

After our exclusion of previously established scoring tools, a quality assessment of 208 ML models, involving 17 types, was conducted. In total, 24% (50/208) of these models originated from case-control studies, which introduced a high risk of bias in study participant selection. Regarding predictive factors, 1% (2/208) of the models were linked to a high risk of bias owing to the use of outcome information. Regarding outcome assessment, as both CA and prognosis outcomes were clearly defined using standard definitions, no additional predictive factors were required, resulting in a low risk of bias in outcome assessment. The included ML models were primarily derived from large-sample statistical analyses; however, 9.1% (19/208) of the models were based on a very small number of cases, with an event per variable value of <10. In addition, inappropriate deletion methods were applied to address missing data in 65.4% (136/208) of the models, and only univariate analysis was used to screen for predictive factors in 22.6% (47/208) of the models, ultimately resulting in a high risk of bias for 76.9% (160/208) of the models in the domain of statistical analysis, as detailed in Figure 2.

### Meta-Analysis

#### CA Occurrence

A meta-analysis of ML models for predicting CA occurrence in the training set was conducted through a random-effects model. The analysis revealed a C-index of 0.84 (95% CI 0.82-0.86; 38/208, 18.3% of the models), sensitivity of 0.78 (95% CI 0.70-0.84; 34/208, 16.3% of the models), and specificity of 0.84 (95% CI 0.80-0.88; 34/208, 16.3% of the models). Similarly, a meta-analysis of ML models for predicting CA occurrence in the validation set was conducted, yielding a C-index of 0.89 (95% CI 0.87-0.91; 52/208, 25% of the models), sensitivity of 0.83 (95% CI 0.78-0.87; 43/208, 20.7% of the models), and specificity of 0.93 (95% CI 0.88-0.96; 43/208, 20.7% of the models; Multimedia Appendices 6-13).

Because of the diverse sources of modeling data from both balanced and imbalanced datasets and the variety of models, a subgroup analysis was conducted based on the data model type. The detailed results are presented in Multimedia Appendices 14-18.

#### Favorable Neurological Outcomes (CPC 1-2)

A meta-analysis of ML models for predicting CPC 1-2 in the training set was conducted using a random-effects model. The results indicated a C-index of 0.90 (95% CI 0.89-0.92; 21/208, 10.1% of the models), sensitivity of 0.72 (95% CI 0.47-0.98; 15/208, 7.2% of the models), and specificity of 0.85 (95% CI 0.79-0.90; 15/208, 7.2% of the models). Similarly, the meta-analysis of ML models for predicting CPC 1-2 in the validation set revealed a C-index of 0.86 (95% CI 0.85-0.87; 69/208, 33.2% of the models), sensitivity of 0.72 (95% CI 0.61-0.81; 44/208, 21.2% of the models), and specificity of 0.79 (95% CI 0.66-0.88; 44/208, 21.2% of the models; Multimedia Appendix 19, and Table S1 and Figures S1-S3 in Multimedia Appendix 20).

It was hypothesized that there might have been differences in CPC 1-2 between patients experiencing IHCA and OHCA. As the real-world data closely resembled balanced datasets, a subgroup analysis was conducted exclusively on the IHCA and OHCA populations. The detailed results are shown in Multimedia Appendix 19, and Table S1 and subgroup analysis report S1 in Multimedia Appendix 20.

#### CA Mortality

A random-effects model was used for the meta-analysis of ML models for predicting CA mortality in the training set. The analysis indicated a C-index of 0.80 (95% CI 0.76-0.84; 14/208, 6.7% of the models), sensitivity of 0.82 (95% CI 0.58-0.94; 7/208, 3.4% of the models), and specificity of 0.76 (95% CI 0.51-0.91; 7/208, 3.4% of the models). Similarly, a meta-analysis of ML models for predicting CA mortality in the validation set revealed a C-index of 0.85 (95% CI 0.82-0.87; 28/208, 13.5% of the models), sensitivity of 0.83 (95% CI 0.79-0.87; 23/208, 11.1% of the models), and specificity of 0.79 (95% CI 0.74-0.83; 23/208, 11.1% of the models; Tables S2-S3 and Figures S4-S6 in Multimedia Appendix 20).

It was thought that there might have been differences in mortality rates between patients experiencing IHCA and OHCA. Given that the real-world data closely resembled balanced datasets, a subgroup analysis was conducted exclusively on the IHCA and OHCA populations. The detailed analysis results are provided in Tables S2-S3 and subgroup analysis report S2 in Multimedia Appendix 20.

#### ROSC Analysis

A meta-analysis of ML models for predicting ROSC following CA in the training set was conducted using a random-effects model. The analysis yielded a C-index of 0.83 (95% CI 0.79-0.88; 10/208, 4.8% of the models), sensitivity of 0.52 (95% CI 0.31-0.73; 8/208, 3.8% of the models), and specificity of 0.91 (95% CI 0.88-0.93; 8/208, 3.8% of the models). Similarly, a meta-analysis of ML models for predicting ROSC in the validation set revealed a C-index of 0.77 (95% CI 0.74-0.80; 13/208, 6.3% of the models), sensitivity of 0.53 (95% CI 0.31-0.74; 6/208, 2.9% of the models), and specificity of 0.88 (95% CI 0.71-0.96; 6/208, 2.9% of the models; Tables S4-S5 and Figures S7-S9 in Multimedia Appendix 20).

It was postulated that there may have been differences in ROSC between patients who experienced IHCA and OHCA. As the real-world data closely resembled balanced datasets, a subgroup analysis was performed solely on the IHCA and OHCA populations. The comprehensive analysis results are provided in Tables S4-S5 and subgroup analysis report S3 in Multimedia Appendix 20.

### Modeling Variables

Modeling variables were extracted and weighted for analysis from the 93 studies on ML models for predicting CA and CPC 1-2. Among the 28 studies on predicting CA, the variables with the highest weights were respiratory rate (n=22, 79%), blood pressure (n=20, 71%), age (n=19, 68%), temperature (n=19, 68%), oxygen saturation (n=15, 54%), and airway (n=9, 32%). Among the 42 studies on predicting CPC 1-2, the results showed that the modeling variables with the highest weights in the IHCA group were rhythm (shockable or nonshockable; 8/10, 80%), age (7/10, 70%), medication use (6/10, 60%), gender (5/10, 50%), and Glasgow Coma Scale (GCS; 5/10, 50%). The modeling variables with the highest weights in the OHCA group were age (25/32, 78%), rhythm (shockable or nonshockable; 24/32, 75%), medication use (18/32, 56%), ROSC (14/32, 44%), gender (12/32, 38%), no-flow time (resuscitation duration; 12/32, 38%), EMS transport (scene interval, arrival time, and response time; 12/32, 38%), defibrillation (11/32, 34%), and GCS (6/32, 19%). The detailed results of the modeling variables and weight analysis are presented in Tables S6 and S7 in Multimedia Appendix 20.

## Discussion

### Summary of the Principal Findings

It was observed that ML has garnered widespread attention among numerous researchers in the management of CA, particularly focusing on early CA risk prediction in both in-hospital and out-of-hospital populations. Our systematic review and meta-analysis demonstrated a relatively favorable predictive value of ML in the validation set for forecasting CA risk, with a C-index of 0.89. Similarly, ML also appeared to exhibit a relatively favorable predictive value for neurological outcomes (CPC 1-2) and mortality in patients who had already experienced CA, with pooled C-indexes of 0.86 and 0.85, respectively. However, in predicting ROSC following CA, ML seemed to display a predictive value comparable to that of traditional scoring tools, with a pooled C-index of 0.77.

### Comparison With Previous Reviews

Currently, in clinical practice, classic early warning scoring tools, including the Cardiac Arrest Risk Triage (CART) score, Modified Early Warning Score, and VitalPAC Early Warning Score, are commonly used for predicting the occurrence of CA. A previous review by Churpek et al [112] found that, among these tools, the CART had the highest accuracy in predicting CA compared to the others. However, the CART had certain limitations, with an area under the curve of 0.83, sensitivity calculated at 0.61 based on the optimal Youden index, and a specificity of 0.84. Moreover, the CART has not been externally validated, and the included population is limited to ward inpatients. Therefore, whether the CART can dynamically monitor the occurrence of CA in real-time clinical events, improve rescue success rates, and enhance patient outcomes requires prospective validation using high-quality, large-sample external data. Our summarized results of the ML models reveal that ML has certain clinical predictive value in forecasting the occurrence of CA and demonstrates relatively favorable accuracy, with an overall C-index of 0.89, sensitivity of 0.83, and specificity of 0.93. Comparatively, this is superior to previous scoring tools, providing a certain clinical basis for the future establishment of more reliable early warning scoring systems for predicting CA.

In a recent review by Carrick et al [113], the accuracy of scoring tools for predicting survival or neurological outcomes following CA, such as the OHCA score, Cardiac Arrest Hospital Prognosis score, and Good Outcome Following Attempted Resuscitation score, was summarized. These 3 tools, which have undergone rigorous clinical validation, exhibited relatively high accuracy, with C-indexes of 0.79, 0.83, and 0.76, respectively. However, our summarized results of ML models suggested that ML seems to exhibit more favorable accuracy, with an overall C-index of 0.86, sensitivity of 0.72, and specificity of 0.79 for predicting favorable neurological outcomes. For predicting CA mortality, ML achieved an overall C-index of 0.85, sensitivity of 0.83, and specificity of 0.79.

Among various ROSC prediction models for CA that have been developed in the current clinical field, the ROSC after CA score developed by Gräsner et al [114] using data from 5471 patients with OHCA from the German Resuscitation Registry has attracted the most attention. It has been externally validated in several European and Asian countries, demonstrating relatively good accuracy, with an area under the curve of 0.736 in a recent large-scale external validation study [115]. However, our summarized results of ML models indicated that the overall C-index of ML was 0.77, with a sensitivity of 0.53 and specificity of 0.88. Comparatively, these ML models did not seem to significantly outperform traditional scoring tools in predicting ROSC outcomes for patients with CA.

### Modeling Variables in ML

In our review, the modeling variables of the discussed models primarily originated from common clinical features. It was found that variables such as respiratory rate, blood pressure, age, temperature, oxygen saturation, and airway were key predictors in existing ML models, and they also constitute critical variables in traditional scoring models [116,117]. Therefore, the impact of these variables on predicting the occurrence of CA is well established. However, these predictors differ to some extent from the findings of recent studies, such as the review by Andersen et al [3], which identified CA risk factors. The review by Andersen et al [3] suggested that age was more associated with post-CA prognosis and reduced survival rates, whereas a history of cardiac diseases such as myocardial infarction, arrhythmias, and heart failure was recognized as the most common risk factor for CA occurrence. Other potential risk factors included the use of certain medications, such as those that prolong the QT interval, opioids, and sedatives. Nonetheless, the review concurred that respiratory function and body temperature also had predictive significance for CA, with early interventions targeting these factors being crucial for achieving reversible outcomes [118].

In the models we reviewed that aimed to predict CPC 1-2 outcomes in patients with IHCA and OHCA, the modeling variables with the highest weight were age, rhythm (shockable or nonshockable), medication use, ROSC, gender, no-flow time (resuscitation duration), defibrillation, EMS transport (scene interval, arrival time, and response time), and GCS. When compared to the review by Sandroni et al [119], which highlighted the predictive value of GCS, biological markers (eg, neuron-specific enolase), and electrophysiological indicators (eg, somatosensory evoked potential) for favorable neurological outcomes, our findings show some differences. In addition, complex variables such as medical imaging might need consideration in clinical practice. In recent years, AI methods have been widely used in medical imaging for identifying disease progression and prognosis, demonstrating superior accuracy and cost-effectiveness compared to traditional clinical feature–based predictive models [120]. Therefore, in the prediction of CA occurrence and prognosis, the high-value variables identified in recent studies, such as electrocardiography [121] and ultrasound [122], are not reflected in traditional scoring tools. This raises the question of whether it is worth further validating these more complex variables and attempting to identify more efficient predictive factors to develop or update risk-scoring tools in the field of CA.

### Clinical Applications of ML

Our study reveals that ML methods appear to outperform traditional scoring tools in predicting the occurrence and progression of CA. Therefore, the development of simple auxiliary tools based on ML theory is recommended to facilitate rapid risk screening of CA for both in-hospital and out-of-hospital patients, enabling timely formulation of appropriate treatment strategies. These ML-based CA prediction models would be particularly beneficial for emergency departments and out-of-hospital response teams. Under the current circumstances, in which emergency departments worldwide are facing challenges of overcrowding, resource limitations, and a high influx of patients who are critically ill [123-125], relying solely on human assessment of CA risk based on various clinical data could pose significant challenges to the efficiency of CA treatment. Furthermore, the complexity and volume of clinical data, including patient demographics, laboratory results, imaging data, and textual notes from health care providers, are continuously increasing. Thus, using ML to analyze large datasets and handle complex variables, such as clinical images, seems to be a more feasible approach [126]. The development of simplified ML prediction tools or intelligent reading systems has the potential to mitigate risks such as treatment delays and poor prognoses in patients with CA in emergency departments. These tools could also enhance health care service quality, reduce human resource costs, and support the formulation of targeted therapeutic strategies. Similarly, ML models that incorporate real-time input of variables such as vital signs, electrocardiograms, and response times for out-of-hospital rescue scenarios can accurately predict positive CA events. This capability aids response teams in avoiding repeated and frequent evaluations, enabling timely decisions on whether to implement preventive therapeutic interventions to avert CA or, in cases of CA occurrence, whether to initiate extracorporeal membrane oxygenation cardiopulmonary resuscitation to improve survival rates [127,128].

In addition, our findings indicate that the balance of data significantly impacts the outcomes of ML model construction in CA-related studies. This effect is particularly pronounced in scenarios in which the outcome metrics exhibit severe imbalance. For instance, in predicting the occurrence of CA in our study, the rationale for the selected predictive factors remained challenging, and the accuracy of the constructed model was often influenced by the overwhelming proportion of negative events [129]. In such cases, the C-index hardly represented the actual outcome prediction accuracy of the model. Therefore, in our study, the sensitivity and specificity of ML models were also summarized [130]. The data balance in the studies we included was primarily addressed using oversampling, but these studies rarely considered validating models constructed from balanced data against imbalanced data. This raises certain doubts regarding the accuracy of existing models constructed based on balanced data when applied to real-world cases of CA. Our study reveals that CA events in the real world are often inherently imbalanced. Therefore, we recommended prioritizing the use of models constructed from real-world data or validating models constructed from balanced data against real-world data to ascertain their true effectiveness [131].

### Ethical Considerations and Model Selection

Although ML models demonstrated relatively satisfactory accuracy in predicting the occurrence and progression of CA in our study, several common challenges inherent to ML modeling should be acknowledged. For instance, compared to traditional scoring tools, ML models rely on general algorithms to generate desired outputs in response to specific input data, a process characterized by less explicit rules [132]. In addition, algorithmic biases may result in unrepresentative datasets [133], and the reliability of model validation remains a concern [134]. These issues underscore the ethical challenges associated with the application of AI in medicine, including result interpretability, algorithmic transparency, predictive fairness, and data privacy [135,136]. These potential ethical concerns are specifically reflected in a patient survey study on the prevention of CA occurrence and development conducted by Maris et al [137]. The study results indicate that, while AI-driven CA treatment decisions offer objective data, the absence of patient involvement and informed consent, along with the interpretability of the model, suggest that the overuse of AI technology may ultimately undermine patient trust in physicians. This, in turn, poses challenges to the current high-quality health care goal of patient-centered care [138] in the field of cardiovascular disease treatment, which is built on shared decision-making [139], respect for patient autonomy, and mutual trust. Therefore, in the high-risk and critical treatment of CA, it seems that physicians should continue to make final decisions in collaboration with ML models based on evidence-based clinical experience and the values of the patients.

Therefore, based on ethical considerations, the choice of different models during research remains challenging as model interpretability and accuracy are factors that need to be considered comprehensively during model construction [140]. Selecting models with higher interpretability, such as LR, Cox regression, or DTs, can facilitate better communication, interaction, and trust between health care providers and patients. However, these models may have limited predictive value for certain outcome events [141]. On the other hand, models whose interpretability is poorer, including neural networks, support vector machines (SVMs), and Extreme Gradient Boosting, often perform exceptionally well in predicting outcomes [142]. At this point, it may become necessary to grant patients and their families greater rights to information and autonomy, enabling their active participation in medical decision-making. In our research, LR was the most frequently used model type as it facilitated the development of predictive nomograms, which are simple and easily applicable tools.

Among the 17 ML models that we included, artificial neural networks, RF, and LR appeared to demonstrate relatively ideal predictive value in forecasting the occurrence of CA and were more frequently used by clinical researchers. In predicting neurological outcomes, our study found that LR remained the model most commonly selected by clinicians, followed by DT, RF, SVM, and others. If we aim to develop a simplified predictive scoring scale to assist in clinical practice, priority may be given to using LR for its development and subsequent updates. This preference arises because, according to our research findings, LR demonstrates relatively satisfactory accuracy and facilitates the construction of straightforward and practical predictive nomograms [143,144]. Furthermore, considering the interpretability of models is essential in real-world clinical practice. However, if the objective is to develop auxiliary applications for disease surveillance and prediction in clinical settings, alternative, more complex models may be considered. For example, models such as neural networks, SVM, and Extreme Gradient Boosting, which demonstrated higher accuracy in our study, could be appropriate choices. On the other hand, when dealing with image-based features, such as medical imaging or electrocardiograms, it may be necessary to use models with lower interpretability, such as those based on deep learning [145], rather than confining the analysis to commonly used clinical features with stronger interpretability.

### Prospects

In addition, we observed a minimal number of studies that constructed models based on artificial neural networks and ensemble learning, which exhibited highly favorable results, indicating that further validation of these models may be required in future research. Currently, in clinical practice, there is an increasing preference for using simple scoring tools based on interpretable clinical features. While opting for such tools may reduce the ethical dilemmas encountered in clinical settings, relying solely on traditional methods and highly interpretable clinical indicators, such as the Delphi method, during the development of these scoring tools seems to introduce significant bias into the constructed models. Therefore, we considered using multicenter real-world big data, using ML approaches, and incorporating a broader range of cases and clinical features to construct interpretable scoring tools and promote their application. Regarding the processing of clinical image features, our expectation lies in the development of intelligent reading tools based on deep learning methods. Nonetheless, in our study, there was limited research on deep learning based on medical imaging and ultrasounds, particularly in the field of CA, where such research remains relatively underexplored. Therefore, future research on CA should actively explore the integration of medical imaging and ultrasonography. In selecting datasets and algorithms for the development of AI prediction models, it is crucial to rigorously investigate and address the ethical shortcomings of AI applications in health care. Efforts should be made to minimize the influence of individual characteristics such as gender, race, skin color, and socioeconomic status, ensuring that population representation and sample size are carefully considered. Sufficient numbers and the quality of representative populations should be selected from diverse regions, ethnicities, and age groups to establish standardized big data models, thereby maximizing the potential of AI technologies [146,147]. It is essential to adopt a multifaceted, interdisciplinary approach; strengthen data protection systems to prevent the leakage of patient information; and conduct extensive reviews to avoid biases [148], ultimately preventing unfairness toward individuals or patient populations in the development of intelligent diagnostic or predictive tools for CA [149,150].

### Advantages and Limitations of This Study

Our study has 3 strengths. First, it represents the first attempt to summarize evidence comparing ML models and scoring tools in predicting the occurrence and prognosis of CA, thereby providing an evidence-based foundation for the subsequent clinical update and development of new scoring tools or AI early warning systems in the field of CA. Second, our study encompassed 93 original studies with large sample sizes, covering 14 countries and involving 5,729,721 patients, significantly enhancing the strength of our evidence. Third, we conducted a detailed discussion of the accuracy of different models on balanced and imbalanced data. However, this study also had the following limitations. First, most of the original studies on the prediction of CA occurrence (28/93, 30%) constructed models based solely on imbalanced data without validating them on balanced data. Second, many model validation processes primarily used internal validation through random sampling, lacking external multicenter validation to examine their generalizability. Third, due to potential differences in the predictive performance of different models for outcome events, despite our in-depth discussion of various ML models and datasets, the limited number of studies on certain ML models restricted our ability to interpret the results of ML applications in CA more comprehensively. Fourth, due to the small number of included studies, we did not strictly distinguish between IHCA and OHCA populations when summarizing the predictors of CA. Fifth, as this review only included English-language original studies, there may be potential language bias.

### Conclusions

Current traditional scoring tools have demonstrated relatively ideal efficacy in predicting the occurrence and prognosis of CA. On the basis of this review, ML appeared to offer greater advantages in predicting the occurrence of CA, neurological functional prognosis, and mortality outcomes. However, for predicting outcomes associated with ROSC after CA, ML models did not seem to significantly outperform traditional models. Therefore, in future studies on CA, researchers may explore the systematic updating of traditional scoring tools based on the superior performance of ML in specific outcomes. This approach would enable the implementation of AI-driven enhancements within complex and diverse clinical data, thereby assisting clinicians in monitoring and providing early warnings for multiple predictive factors. For outcomes that are still unpredictable, multicenter large-sample studies are warranted.

## Appendix

Multimedia Appendix 1 PRISMA (Preferred Reporting Items for Systematic Reviews and Meta-Analyses) checklist.

Multimedia Appendix 2 Literature search strategy in PubMed.

Multimedia Appendix 3 Literature search strategy in the Cochrane Library.

Multimedia Appendix 4 Literature search strategy in Embase.

Multimedia Appendix 5 Literature search strategy in Web of Science.

Multimedia Appendix 6 Meta-analysis results for the C-index of predictive models of in-hospital cardiac arrest risk.

Multimedia Appendix 7 Meta-analysis results for the sensitivity and specificity of predictive models for in-hospital cardiac arrest risk.

Multimedia Appendix 8 Forest plot of the C-index meta-analysis of prediction models of in-hospital cardiac arrest risk in the training set.

Multimedia Appendix 9 Forest plot of the C-index meta-analysis of prediction models of in-hospital cardiac arrest risk in the validation set.

Multimedia Appendix 10 Forest plot of the sensitivity meta-analysis of predictive models of in-hospital cardiac arrest risk in the training set.

Multimedia Appendix 11 Forest plot of the sensitivity meta-analysis of predictive models of in-hospital cardiac arrest risk in the validation set.

Multimedia Appendix 12 Forest plot of the specificity meta-analysis of predictive models of in-hospital cardiac arrest risk in the training set.

Multimedia Appendix 13 Forest plot of the specificity meta-analysis of predictive models of in-hospital cardiac arrest risk in the validation set.

Multimedia Appendix 14 Subgroup analysis for predicting cardiac arrest occurrence.

Multimedia Appendix 15 Meta-analysis results for the C-index of predictive models of in-hospital cardiac arrest risk in balanced datasets.

Multimedia Appendix 16 Meta-analysis results for the sensitivity and specificity of risk prediction models for in-hospital cardiac arrest in balanced datasets.

Multimedia Appendix 17 Meta-analysis results for the C-index of prediction models for in-hospital cardiac arrest risk in imbalanced datasets.

Multimedia Appendix 18 Meta-analysis results for the sensitivity and specificity of risk prediction models for in-hospital cardiac arrest in imbalanced datasets.

Multimedia Appendix 19 Meta-analysis results for the C-index of predictive models of favorable neurological function (good cerebral performance category score 1-2).

Multimedia Appendix 20 Tables/figures/subgroup analysis results.

## Abbreviations

AI: artificial intelligence
CA: cardiac arrest
CART: Cardiac Arrest Risk Triage
CPC 1-2: good cerebral performance category score 1 to 2
DT: decision tree
EMS: emergency medical services
GCS: Glasgow Coma Scale
IHCA: in-hospital cardiac arrest
LR: logistic regression
ML: machine learning
OHCA: out-of-hospital cardiac arrest
PRISMA: Preferred Reporting Items for Systematic Reviews and Meta-Analyses
PROBAST: Prediction Model Risk of Bias Assessment Tool
RF: random forest
ROSC: return of spontaneous circulation
SVM: support vector machine

## Data Availability

All data generated or analyzed during this study are included in this published article and its supplementary information files.
