# Supplementary material for: Application of Machine Learning for Patients With Cardiac Arrest: Systematic Review and Meta-Analysis
Source: J Med Internet Res. 2025 Mar 10;27:e67871. doi: 10.2196/67871 (PMC11933771; doi:10.2196/67871)
Supplement: Multimedia Appendix 3 [file jmir_v27i1e67871_app3.docx]

**Multimedia Appendix 3. Literature search strategy in the Cochrane Library.**

| Search number | Query | Results |
| --- | --- | --- |
| #1 | MeSH descriptor: [Emergency Service, Hospital] explode all trees | 3314 |
| #2 | (‘Emergency Service, Hospital’ or ‘Hospital Emergency Services’ or ‘Emergency Hospital Service’ or ‘Emergency Hospital Services’ or ‘Hospital Service Emergency’ or ‘Hospital Service Emergencies’ or ‘Hospital Emergency Service’ or ‘Emergency Units’ or ‘Emergency Unit’ or ‘Accident and Emergency Department’ or ‘Emergency Ward’ or ‘Emergency Wards’ or ‘Emergency Departments’ or ‘Emergency Department’ or ‘Emergency Room’ or ‘Emergency Rooms’ or ‘Emergency Outpatient Unit’ or ‘Emergency Outpatient Units’ or ‘A and E department’ or ‘A and E unit’ or ‘A and E ward’ or ‘A-E department’):ti,ab,kw | 36100 |
| #3 | MeSH descriptor: [Machine Learning] explode all trees | 880 |
| #4 | (‘machine learning’ or ‘Transfer Learning ’ or ‘Deep learning ’ or ‘Ensemble Learning ’ or ‘artificial intelligence ’ or ‘Prediction model ’ or ‘random forest ’ or ‘neural network ’ or ‘neural networks’ or ‘CNN ’ or ‘Support vector machine ’ or ‘SVM ’ or ‘Gradient Boosting Machine ’ or ‘Nomogram ’ or ‘XGBoost ’ or ‘Adaboost ’ or ‘Decision tree ’ or ‘ResNet-50 ’ or ‘ResNet ’ or ‘Naive Bayesian ’ or ‘Multilayer perceptron ’ or ‘Bayesian network ’ or ‘K-Nearest Neighbor’):ti,ab,kw | 15489 |
| #5 | MeSH descriptor: [Heart Arrest] explode all trees | 2864 |
| #6 | (‘Heart Arrest’ or ‘Cardiac Arrest ’ or ‘Asystole ’ or ‘Asystoles ’ or ‘Cardiopulmonary Arrest ’ or ‘asystolia’):ti,ab,kw | 6656 |
| #7 | #1 or #2 | 36260 |
| #8 | #3 or #4 | 15489 |
| #9 | #5 or #6 | 7390 |
| #10 | #7 and #8 and #9 | 18 |
