# Supplementary material for: Application of Machine Learning for Patients With Cardiac Arrest: Systematic Review and Meta-Analysis
Source: J Med Internet Res. 2025 Mar 10;27:e67871. doi: 10.2196/67871 (PMC11933771; doi:10.2196/67871)
Supplement: Multimedia Appendix 5 [file jmir_v27i1e67871_app5.docx]

**Multimedia Appendix 5. Literature search strategy in Web of Science.**

| Search number | Query | Results |
| --- | --- | --- |
| #1 | "‘Emergency Service, Hospital’ or ‘Hospital Emergency Services’ or ‘Emergency Hospital Service’ or ‘Emergency Hospital Services’ or ‘Hospital Service Emergency’ or ‘Hospital Service Emergencies’ or ‘Hospital Emergency Service’ or ‘Emergency Units’ or ‘Emergency Unit’ or ‘Accident and Emergency Department’ or ‘Emergency Ward’ or ‘Emergency Wards’ or ‘Emergency Departments’ or ‘Emergency Department’ or ‘Emergency Room’ or ‘Emergency Rooms’ or ‘Emergency Outpatient Unit’ or ‘Emergency Outpatient Units’ or ‘A and E department’ or ‘A and E unit’ or ‘A and E ward’ or ‘A-E department’ (Topic) AND ‘machine learning’ or ‘Transfer Learning ’ or ‘Deep learning ’ or ‘Ensemble Learning ’ or ‘artificial intelligence ’ or ‘Prediction model ’ or ‘random forest ’ or ‘neural network ’ or ‘neural networks’ or ‘CNN ’ or ‘Support vector machine ’ or ‘SVM ’ or ‘Gradient Boosting Machine ’ or ‘Nomogram ’ or ‘XGBoost ’ or ‘Adaboost ’ or ‘Decision tree ’ or ‘ResNet-50 ’ or ‘ResNet ’ or ‘Naive Bayesian ’ or ‘Multilayer perceptron ’ or ‘Bayesian network ’ or ‘K-Nearest Neighbor’ (Topic) AND ‘Heart Arrest’ or ‘Cardiac Arrest ’ or ‘Asystole ’ or ‘asystole ’ or ‘Cardiopulmonary Arrest ’ or ‘asystolic’ (Topic) and Preprint Citation Index (Exclude – Database) " | 347 |
