# Supplementary material for: Application of Machine Learning for Patients With Cardiac Arrest: Systematic Review and Meta-Analysis
Source: J Med Internet Res. 2025 Mar 10;27:e67871. doi: 10.2196/67871 (PMC11933771; doi:10.2196/67871)
Supplement: Multimedia Appendix 6 [file jmir_v27i1e67871_app6.docx]

**Multimedia Appendix 6. Meta-analysis results for the C-index of predictive models of in-hospital cardiac arrest risk.**

| Model type | Training set | | | | Validation set | | | |
| --- | --- | --- | --- | --- | --- | --- | --- | --- |
|  | Events | Sample size | n | C-index(95%CI) | Events | Sample size | n | C-index(95%CI) |
| Machine learning |  |  |  |  |  |  |  |  |
| RF(Random Forest) | 41,977 | 952,040 | 6 | 0.89(0.86-0.93) | 50,532 | 307,803 | 11 | 0.89(0.84-0.94) |
| DT(Decision Tree) | 1,849 | 90,667 | 4 | 0.76(0.68-0.85) | 1,056 | 78,740 | 3 | 0.87(0.76-0.98) |
| SVM(Support Vector Machine) | 1,437 | 2,873 | 1 | 0.83(0.82-0.84) | 402 | 1,644 | 2 | 0.86(0.84-0.89) |
| XGBoost | 2,391 | 477,571 | 3 | 0.98(0.96-0.99) | 49,198 | 472,659 | 6 | 0.91(0.89-0.93) |
| LR(Logistic Regression) | 4,962 | 1,241,304 | 11 | 0.86(0.83-0.90) | 4,664 | 641,036 | 16 | 0.87(0.81-0.92) |
| NB(Naïve Bayes) | 124 | 43,569 | 1 | 0.66(0.61-0.71) | NA | NA | NA | NA |
| DL(Deep Learning) | 355 | 87,624 | 3 | 0.63(0.47-0.79) | 554 | 267,308 | 5 | 0.89(0.86-0.92) |
| AdaBoost | 124 | 43,569 | 1 | 0.57(0.52-0.62) | NA | NA | NA | NA |
| ANN(Artificial Neural Network) | 3,481 | 998,807 | 8 | 0.86(0.83-0.89) | 4504 | 439,601 | 9 | 0.94(0.91-0.96) |
| Overall | 56,700 | 3,938,024 | 38 | 0.84(0.82-0.86) | 110,910 | 2,208,791 | 52 | 0.89(0.87-0.91) |
| Scoring system |  |  |  |  |  |  |  |  |
| NEWS |  |  |  |  | 1,725 | 466,817 | 7 | 0.79(0.74-0.84) |
| MEWS |  |  |  |  | 3,314 | 1,256,197 | 11 | 0.78(0.74-0.81) |
| NEWS2 |  |  |  |  | 159 | 79,116 | 1 | 0.68(0.68-0.68) |
| EDICAS |  |  |  |  | 240 | 145,557 | 1 | 0.88(0.86-0.90) |
| REMS |  |  |  |  | 240 | 145,557 | 1 | 0.83(0.80-0.86) |
| PSS |  |  |  |  | 52 | 1,025 | 2 | 0.84(0.72-0.97) |
| DSS |  |  |  |  | 52 | 1,025 | 2 | 0.78(0.70-0.87) |
| Overall |  |  |  |  | 5,782 | 2,095,294 | 25 | 0.79(0.75-0.82） |

Note: NEWS: National Early Warning Score, MEWS: Modified early warning score, NEWS2: National Early Warning Score 2, EDICAS: Emergency Department In-hospital Cardiac Arrest Score, REMS: Rapid Emergency Medicine Score, PSS: Proposed scoring system, DSS: Distance scoring system.
