# Supplementary material for: Application of Machine Learning for Patients With Cardiac Arrest: Systematic Review and Meta-Analysis
Source: J Med Internet Res. 2025 Mar 10;27:e67871. doi: 10.2196/67871 (PMC11933771; doi:10.2196/67871)
Supplement: Multimedia Appendix 7 [file jmir_v27i1e67871_app7.docx]

**Multimedia Appendix 7. Meta-analysis results for the sensitivity and specificity of predictive models for in-hospital cardiac arrest risk.**

| Model type | Training set | | | Validation set | | |
| --- | --- | --- | --- | --- | --- | --- |
|  | n | Sensitivity (95%CI) | Specificity (95%CI) | n | Sensitivity (95%CI) | Specificity (95%CI) |
| Machine learning |  |  |  |  |  |  |
| RF(Random Forest) | 6 | 0.81(0.70-0.89) | 0.86(0.79-0.91) | 10 | 0.84(0.65-0.94) | 0.94(0.82-0.98) |
| DT(Decision Tree) | 4 | 0.72(0.54-0.85) | 0.77(0.66-0.86) | 3 | 0.57-0.89 | 0.77-1.00 |
| SVM(Support Vector Machine) | 1 | 0.99 | 0.50 | 2 | 0.81-1.00 | 0.53-0.72 |
| XGBoost | 3 | 0.90-0.99 | 0.83-0.99 | 5 | 0.74(0.50-0.89) | 0.98(0.62-1.00) |
| LR(Logistic Regression) | 9 | 0.76(0.69-0.82) | 0.84(0.80-0.87) | 12 | 0.80(0.75-0.84) | 0.84(0.77-0.89) |
| NB(Naïve Bayes) | 1 | 0.67 | 0.62 | NA | NA | NA |
| KNN(K-Nearest Neighbor) | 3 | 0.42-0.56 | 0.70-0.90 | 5 | 0.78(0.67-0.85) | 0.87(0.79-0.92) |
| AdaBoost | 1 | 0.53 | 0.60 | NA | NA | NA |
| ANN(Artificial Neural Network) | 6 | 0.63(0.52-0.74) | 0.86(0.76-0.92) | 6 | 0.90(0.86-0.92) | 0.98(0.93-1.00) |
| Overall | 34 | 0.78(0.70-0.84) | 0.84(0.80-0.88) | 43 | 0.83(0.78-0.87) | 0.93(0.88-0.96) |
| Scoring system |  |  |  |  |  |  |
| NEWS |  |  |  | 7 | 0.72(0.63-0.80) | 0.76(0.69-0.82) |
| MEWS |  |  |  | 10 | 0.67(0.61-0.73) | 0.79(0.70-0.86) |
| NEWS2 |  |  |  | 1 | 0.26 | 0.91 |
| EDICAS |  |  |  | 1 | 0.85 | 0.75 |
| REMS |  |  |  | 1 | 0.85 | 0.67 |
| PSS |  |  |  | 1 | 0.77 | 0.63 |
| DSS |  |  |  | 1 | 0.77 | 0.63 |
| Overall |  |  |  | 22 | 0.70(0.63-0.76) | 0.77(0.72-0.82) |

Note: NEWS: National Early Warning Score, MEWS: Modified early warning score, NEWS2: National Early Warning Score 2, EDICAS: Emergency Department In-hospital Cardiac Arrest Score, REMS: Rapid Emergency Medicine Score, PSS: Proposed scoring system, DSS: Distance scoring system.
