# Supplementary material for: Application of Machine Learning for Patients With Cardiac Arrest: Systematic Review and Meta-Analysis
Source: J Med Internet Res. 2025 Mar 10;27:e67871. doi: 10.2196/67871 (PMC11933771; doi:10.2196/67871)
Supplement: Multimedia Appendix 9 [file jmir_v27i1e67871_app9.docx]

**Multimedia Appendix 9. Forest plot of the C-index meta-analysis of prediction models of in-hospital cardiac arrest risk in the validation set.**

**
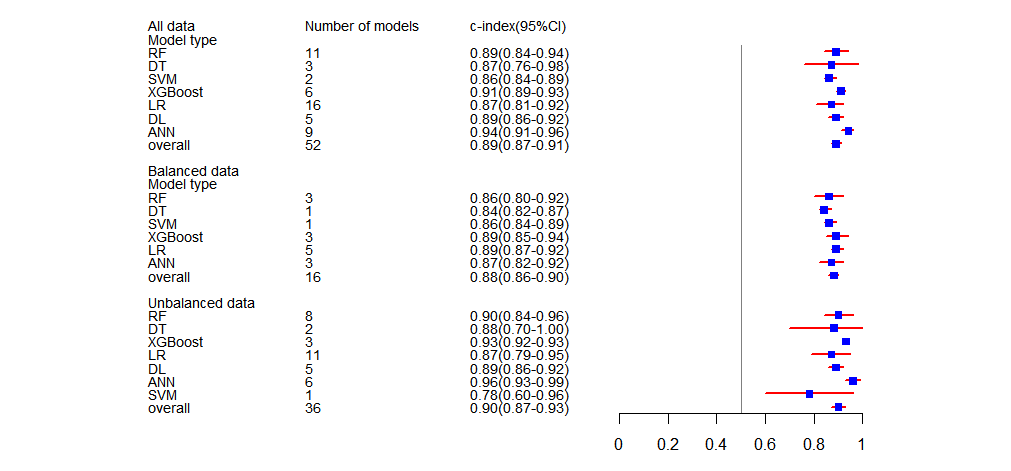
**
