# Supplementary material for: Application of Machine Learning for Patients With Cardiac Arrest: Systematic Review and Meta-Analysis
Source: J Med Internet Res. 2025 Mar 10;27:e67871. doi: 10.2196/67871 (PMC11933771; doi:10.2196/67871)
Supplement: Multimedia Appendix 12 [file jmir_v27i1e67871_app12.docx]

**Multimedia Appendix 12. Forest plot of the specificity meta-analysis of predictive models of in-hospital cardiac arrest risk in the training set.**

**
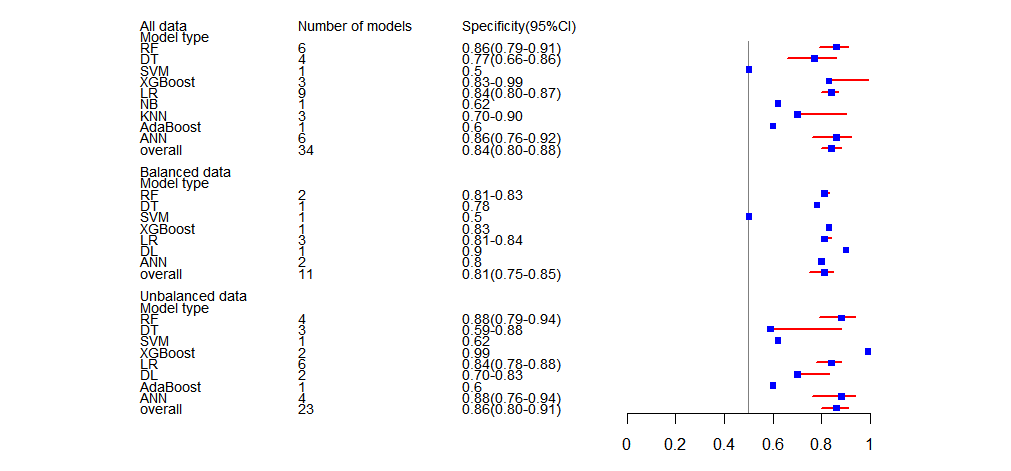
**
