# Supplementary material for: Application of Machine Learning for Patients With Cardiac Arrest: Systematic Review and Meta-Analysis
Source: J Med Internet Res. 2025 Mar 10;27:e67871. doi: 10.2196/67871 (PMC11933771; doi:10.2196/67871)
Supplement: Multimedia Appendix 14 [file jmir_v27i1e67871_app14.docx]

**Multimedia Appendix 14. Subgroup analysis for predicting cardiac arrest occurrence.**

In the real-world data used for predicting the occurrence of CA, the pooled incidence of cardiac arrest was approximately 0.52% (21,500/4,119,195). This observation indicated that the real-world data represented a severely imbalanced dataset. Therefore, we considered both balanced and imbalanced data in the subgroup analysis results of machine learning models. For balanced data, we performed a meta-subgroup analysis using a random-effects model on the machine learning models predicting CA occurrence in the training set. The analysis revealed that the C-index, sensitivity, and specificity were 0.85 (95% CI: 0.82-0.88, n=13), 0.78 (95% CI: 0.63-0.89, n=11), and 0.81 (95% CI: 0.75-0.85, n=11), respectively. Similarly, using the random-effects model for the validation set, the meta-subgroup analysis showed a C-index, sensitivity, and specificity of 0.88 (95% CI: 0.86-0.90, n=16), 0.72 (95% CI: 0.49-0.95, n=8), and 0.79 (95% CI: 0.68-0.91, n=8), respectively. For imbalanced data, a random-effects model was also employed for meta-subgroup analysis on the training set, revealing a C-index, sensitivity, and specificity of 0.83 (95% CI: 0.81-0.85, n=25), 0.77 (95% CI: 0.68-0.84, n=23), and 0.86 (95% CI: 0.80-0.91, n=23), respectively. The validation set analysis showed a C-index, sensitivity, and specificity of 0.90 (95% CI: 0.87-0.93, n=36), 0.83 (95% CI: 0.79-0.87, n=35), and 0.93 (95% CI: 0.88-0.96, n=35), respectively.
