# Supplementary material for: Application of Machine Learning for Patients With Cardiac Arrest: Systematic Review and Meta-Analysis
Source: J Med Internet Res. 2025 Mar 10;27:e67871. doi: 10.2196/67871 (PMC11933771; doi:10.2196/67871)
Supplement: Multimedia Appendix 15 [file jmir_v27i1e67871_app15.docx]

**Multimedia Appendix 15. Meta-analysis results for the C-index of predictive models of in-hospital cardiac arrest risk in balanced datasets.**

| Model type | Training set | | | | Validation set | | | |
| --- | --- | --- | --- | --- | --- | --- | --- | --- |
|  | Events | Sample size | n | C-index(95%CI) | Events | Sample size | n | C-index(95%CI) |
| Machine learning |  |  |  |  |  |  |  |  |
| RF(Random Forest) | 1,544 | 3,359 | 2 | 0.85(0.66-1.00) | 48,239 | 80,655 | 3 | 0.86(0.80-0.92) |
| DT(Decision Tree) | 1,437 | 2,873 | 1 | 0.87(0.86-0.88) | 359 | 719 | 1 | 0.84(0.82-0.87) |
| SVM(Support Vector Machine) | 1,437 | 2,873 | 1 | 0.83(0.82-0.84) | 359 | 719 | 1 | 0.86(0.84-0.89) |
| XGBoost | 1,437 | 2,873 | 1 | 0.94(0.93-0.95) | 47,829 | 79,835 | 3 | 0.89(0.85-0.94) |
| LR(Logistic Regression) | 1,667 | 3,704 | 4 | 0.89(0.86-0.92) | 899 | 1,742 | 5 | 0.89(0.87-0.92) |
| DL(Deep Learning) | 107 | 486 | 1 | 0.77(0.72-0.82) | NA | NA | NA | NA |
| ANN(Artificial Neural Network) | 214 | 972 | 3 | 0.76(0.49-1.00) | 410 | 820 | 3 | 0.87(0.82-0.92) |
| Overall | 7,843 | 17,140 | 13 | 0.85(0.82-0.88) | 98,095 | 164,490 | 16 | 0.88(0.86-0.90) |
| Scoring system |  |  |  |  |  |  |  |  |
| NEWS |  |  |  |  | 107 | 486 | 1 | 0.57(0.51-0.63) |
| MEWS |  |  |  |  | NA | NA | 2 | 0.73(0.67-0.79) |
| PSS |  |  |  |  | NA | NA | 1 | 0.90(0.86-0.95) |
| DSS |  |  |  |  | NA | NA | 1 | 0.82(0.77-0.88) |
| Overall |  |  |  |  | 107 | 486 | 5 | 0.75(0.70-0.81) |

Note: NEWS: National Early Warning Score, MEWS: Modified early warning score, PSS: Proposed scoring system, DSS: Distance scoring system.
