# Supplementary material for: Application of Machine Learning for Patients With Cardiac Arrest: Systematic Review and Meta-Analysis
Source: J Med Internet Res. 2025 Mar 10;27:e67871. doi: 10.2196/67871 (PMC11933771; doi:10.2196/67871)
Supplement: Multimedia Appendix 16 [file jmir_v27i1e67871_app16.docx]

**Multimedia Appendix 16. Meta-analysis results for the sensitivity and specificity of risk prediction models for in-hospital cardiac arrest in balanced datasets.**

| Model type | Training set | | | Validation set | | |
| --- | --- | --- | --- | --- | --- | --- |
|  | n | Sensitivity (95%CI) | Specificity (95%CI) | n | Sensitivity (95%CI) | Specificity (95%CI) |
| Machine learning |  |  |  |  |  |  |
| RF(Random Forest) | 2 | 0.58-0.89 | 0.81-0.83 | 2 | 0.46-0.73 | 0.83-0.98 |
| DT(Decision Tree) | 1 | 0.90 | 0.78 | 1 | 0.86 | 0.77 |
| SVM(Support Vector Machine) | 1 | 0.99 | 0.50 | 1 | 1 | 0.53 |
| XGBoost | 1 | 0.90 | 0.83 | 2 | 0.23-0.87 | 0.82-1.00 |
| LR(Logistic Regression) | 3 | 0.68-0.79 | 0.81-0.84 | 2 | 0.77-0.85 | 0.77-0.84 |
| DL(Deep Learning) | 1 | 0.56 | 0.90 | NA | NA | NA |
| ANN(Artificial Neural Network) | 2 | 0.41-0.45 | 0.80 | NA | NA | NA |
| Overall | 11 | 0.78(0.63-0.89) | 0.81(0.75-0.85) | 8 | 0.72(0.49-0.95) | 0.79(0.68-0.91) |
| Scoring system |  |  |  |  |  |  |
| NEWS |  |  |  | 1 | 0.5 | 0.677 |
| Overall |  |  |  | 1 | 0.5 | 0.677 |

Note: NEWS: National Early Warning Score.
