# Supplementary material for: Application of Machine Learning for Patients With Cardiac Arrest: Systematic Review and Meta-Analysis
Source: J Med Internet Res. 2025 Mar 10;27:e67871. doi: 10.2196/67871 (PMC11933771; doi:10.2196/67871)
Supplement: Multimedia Appendix 17 [file jmir_v27i1e67871_app17.docx]

**Multimedia Appendix 17. Meta-analysis results for the C-index of prediction models for in-hospital cardiac arrest risk in imbalanced datasets.**

| Model type | Training set | | | | Validation set | | | |
| --- | --- | --- | --- | --- | --- | --- | --- | --- |
|  | Events | Sample size | n | C-index(95%CI) | Events | Sample size | n | C-index(95%CI) |
| Machine learning |  |  |  |  |  |  |  |  |
| RF(Random Forest) | 2,653 | 948,681 | 4 | 0.91(0.87-0.95) | 2,293 | 227,148 | 8 | 0.90(0.84-0.96) |
| DT(Decision Tree) | 412 | 87,794 | 3 | 0.72(0.58-0.86) | 697 | 78,021 | 2 | 0.88(0.70-1.00) |
| NB(Naïve Bayes) | 124 | 43,569 | 1 | 0.66(0.61-0.71) | NA | NA | NA | NA |
| XGBoost | 954 | 474,698 | 2 | 0.99(0.99-1.00) | 1,369 | 392,824 | 3 | 0.93(0.92-0.93) |
| LR(Logistic Regression) | 3,295 | 1,237,600 | 7 | 0.85(0.80-0.90) | 3,765 | 639,294 | 11 | 0.87(0.79-0.95) |
| DL(Deep Learning) | 248 | 87,138 | 2 | 0.56(0.42-0.70) | 554 | 267,308 | 5 | 0.89(0.86-0.92) |
| AdaBoost | 124 | 43,569 | 1 | 0.57(0.52-0.62) | NA | NA | NA | NA |
| ANN(Artificial Neural Network) | 3,267 | 997,835 | 5 | 0.90(0.87-0.93) | 4,094 | 438,781 | 6 | 0.96(0.93-0.99) |
| SVM(Support Vector Machine) | NA | NA | NA | NA | 43 | 925 | 1 | 0.78(0.60-0.96) |
| Overall | 11,077 | 3,920,884 | 25 | 0.83(0.81-0.85) | 12,815 | 2,044,301 | 36 | 0.90(0.87-0.93) |
| Scoring system |  |  |  |  |  |  |  |  |
| NEWS |  |  |  |  | 1,618 | 466,331 | 6 | 0.82(0.78-0.87) |
| MEWS |  |  |  |  | 3,314 | 1,256,197 | 9 | 0.80(0.77-0.82) |
| NEWS2 |  |  |  |  | 159 | 79,116 | 1 | 0.68(0.68-0.68) |
| EDICAS |  |  |  |  | 240 | 145,557 | 1 | 0.88(0.86-0.90) |
| REMS |  |  |  |  | 240 | 145,557 | 1 | 0.83(0.80-0.86) |
| PSS |  |  |  |  | 52 | 1,025 | 1 | 0.77(0.71-0.84) |
| DSS |  |  |  |  | 52 | 1,025 | 1 | 0.73(0.65-0.82) |
| Overall |  |  |  |  | 5,675 | 2,094,808 | 20 | 0.80(0.75-0.84) |

Note: NEWS: National Early Warning Score, MEWS: Modified early warning score, NEWS2: National Early Warning Score 2, EDICAS: Emergency Department In-hospital Cardiac Arrest Score, REMS: Rapid Emergency Medicine Score, PSS: Proposed scoring system, DSS: Distance scoring system.
