# Supplementary material for: Application of Machine Learning for Patients With Cardiac Arrest: Systematic Review and Meta-Analysis
Source: J Med Internet Res. 2025 Mar 10;27:e67871. doi: 10.2196/67871 (PMC11933771; doi:10.2196/67871)
Supplement: Multimedia Appendix 18 [file jmir_v27i1e67871_app18.docx]

**Multimedia Appendix 18. Meta-analysis results for the sensitivity and specificity of risk prediction models for in-hospital cardiac arrest in imbalanced datasets.**

| Model type | Training set | | | Validation set | | |
| --- | --- | --- | --- | --- | --- | --- |
|  | n | Sensitivity (95%CI) | Specificity (95%CI) | n | Sensitivity (95%CI) | Specificity (95%CI) |
| Machine learning |  |  |  |  |  |  |
| RF(Random Forest) | 4 | 0.83(0.71-0.91) | 0.88(0.79-0.94) | 8 | 0.89（0.69-0.96） | 0.94（0.78-0.99） |
| DT(Decision Tree) | 3 | 0.50-0.76 | 0.59-0.88 | 2 | 0.57-0.89 | 0.96-1.00 |
| SVM(Support Vector Machine) | 1 | 0.67 | 0.62 | 1 | 0.81 | 0.72 |
| XGBoost | 2 | 0.98-0.99 | 0.99 | 3 | 0.74-0.90 | 0.82-0.93 |
| LR(Logistic Regression) | 6 | 0.77(0.67-0.85) | 0.84(0.78-0.88) | 10 | 0.80(0.74-0.85) | 0.85(0.77-0.90) |
| DL(Deep Learning) | 2 | 0.42-0.55 | 0.70-0.83 | 5 | 0.78(0.67-0.85) | 0.87(0.79-0.92) |
| AdaBoost | 1 | 0.53 | 0.60 | NA | NA | NA |
| ANN(Artificial Neural Network) | 4 | 0.72(0.68-0.76) | 0.88(0.76-0.94) | 6 | 0.90(0.86-0.92) | 0.98(0.93-1.00) |
| Overall | 23 | 0.77(0.68-0.84) | 0.86(0.80-0.91) | 35 | 0.83(0.79-0.87) | 0.93(0.88-0.96) |
| Scoring system |  |  |  |  |  |  |
| NEWS |  |  |  | 6 | 0.75(0.68-0.81) | 0.77(0.70-0.83) |
| MEWS |  |  |  | 10 | 0.67(0.61-0.73) | 0.79(0.70-0.86) |
| NEWS2 |  |  |  | 1 | 0.26 | 0.91 |
| EDICAS |  |  |  | 1 | 0.85 | 0.75 |
| REMS |  |  |  | 1 | 0.85 | 0.67 |
| PSS |  |  |  | 1 | 0.77 | 0.63 |
| DSS |  |  |  | 1 | 0.77 | 0.63 |
| Overall |  |  |  | 21 | 0.71(0.64-0.76) | 0.78(0.72-0.82) |

Note: NEWS: National Early Warning Score, MEWS: Modified early warning score, NEWS2: National Early Warning Score 2, EDICAS: Emergency Department In-hospital Cardiac Arrest Score, REMS: Rapid Emergency Medicine Score, PSS: Proposed scoring system, DSS: Distance scoring system.
