# Supplementary material for: Application of Machine Learning for Patients With Cardiac Arrest: Systematic Review and Meta-Analysis
Source: J Med Internet Res. 2025 Mar 10;27:e67871. doi: 10.2196/67871 (PMC11933771; doi:10.2196/67871)
Supplement: Multimedia Appendix 19 [file jmir_v27i1e67871_app19.docx]

**Multimedia Appendix 19. Meta-analysis results for the C-index of predictive models of favorable neurological function (good cerebral performance category score 1-2).**

| Model type | Training set | | | | Validation set | | | |
| --- | --- | --- | --- | --- | --- | --- | --- | --- |
|  | Events | Sample size | n | C-index(95%CI) | Events | Sample size | n | C-index(95%CI) |
| Machine learning |  |  |  |  |  |  |  |  |
| RF(Random Forest) | 664 | 6,369 | 2 | 0.91(0.86-0.95) | 3,518 | 38,559 | 7 | 0.80(0.74-0.85) |
| DT(Decision Tree) | 5,948 | 311,817 | 3 | 0.87(0.81-0.92) | 2,350 | 86,493 | 8 | 0.78(0.74-0.82) |
| SVM(Support Vector Machine) | NA | NA | NA | NA | 1,640 | 13,188 | 5 | 0.90(0.88-0.92) |
| XGBoost | 105 | 5,739 | 1 | 0.93(0.92-0.93) | 1,025 | 6,725 | 6 | 0.80(0.70-0.90) |
| LR(Logistic Regression) | 2,405 | 19,194 | 11 | 0.90(0.88-0.92) | 8,186 | 32,548 | 23 | 0.88(0.86-0.90) |
| EL(Ensemble Learning) | NA | NA | NA | NA | 212 | 272 | 2 | 0.96(0.91-1.00) |
| DL(Deep Learning) | 5,656 | 149,425 | 1 | 0.968(0.966-0.970) | 8,157 | 174,414 | 9 | 0.90(0.85-0.94) |
| LightGBM | 5,659 | 197,256 | 2 | 0.91(0.91-0.92) | 181 | 841 | 2 | 0.75(0.72-0.78) |
| ANN(Artificial Neural Network) | 75 | 637 | 1 | 1.000(0.89-0.92) | 1,230 | 9,757 | 4 | 0.96(0.92-0.99) |
| KNN(K-Nearest Neighbor) | NA | NA | NA | NA | 181 | 841 | 3 | 0.75(0.62-0.89) |
| Location of CA Occurrence |  |  |  |  |  |  |  |  |
| In-Hospital | 1,787 | 3,631 | 6 | 0.92(0.90-0.95) | 771 | 1,711 | 5 | 0.91(0.88-0.95) |
| Out-of-Hospital | 18,725 | 686,806 | 15 | 0.90(0.88-0.92) | 25,909 | 361,927 | 64 | 0.86(0.85-0.87) |
| Overall | 20,512 | 690,437 | 21 | 0.90(0.89-0.92) | 26,680 | 363,638 | 69 | 0.86(0.85-0.87) |
| Scoring system |  |  |  |  |  |  |  |  |
| CANPS |  |  |  |  | 46 | 159 | 2 | 0.96(0.93-0.99) |
| OCAS |  |  |  |  | 370 | 819 | 2 | 0.83(0.81-0.86) |
| TCAHPS |  |  |  |  | 309 | 713 | 1 | 0.84(0.81-0.87) |
| Overall |  |  |  |  | 725 | 1,691 | 5 | 0.89(0.83-0.95) |

Note: CANPS: Cardiac Original Article Arrest Neurological Prognosis (CANP) Score, OCAS: Out-of-hospital cardiac arrest score, TCAHPS: The Cardiac Arrest Hospital Prognosis score.
