# Supplementary material for: Application of Machine Learning for Patients With Cardiac Arrest: Systematic Review and Meta-Analysis
Source: J Med Internet Res. 2025 Mar 10;27:e67871. doi: 10.2196/67871 (PMC11933771; doi:10.2196/67871)
Supplement: Multimedia Appendix 20 [file jmir_v27i1e67871_app20.docx]

**Table S1. Meta-analysis results for the sensitivity and specificity of models predicting favorable neurological outcomes (good cerebral performance category score 1-2).**

| Model type | Training set | | | Validation set | | |
| --- | --- | --- | --- | --- | --- | --- |
|  | n | Sensitivity(95%CI) | Specificity(95%CI) | n | Sensitivity(95%CI) | Specificity(95%CI) |
| Machine learning |  |  |  |  |  |  |
| RF(Random Forest) | 2 | 0.2-0.83 | 0.86-0.98 | 4 | 0.83(0.65-0.93) | 0.93(0.69-0.99) |
| DT(Decision Tree) | 1 | 1.00 | 0.64 | 4 | 0.88(0.74-0.95) | 0.97(0.80-1.00) |
| SVM(Support Vector Machine) | NA | NA | NA | 3 | 0.63-0.95 | 0.57-0.97 |
| XGBoost | 1 | 0.84 | 0.85 | 2 | 0.66-0.88 | 0.90-0.97 |
| LR(Logistic Regression) | 9 | 0.59(0.33-0.81) | 0.59(0.33-0.81) | 19 | 0.63(0.45-0.78) | 0.58(0.40-0.75) |
| EL(Ensemble Learning) | NA | NA | NA | 2 | 0.65-0.97 | 0.96 |
| KNN(K-Nearest Neighbor) | NA | NA | NA | NA | NA | NA |
| LightGBM | 1 | 0.10 | 1.00 | NA | NA | NA |
| ANN(Artificial Neural Network) | 1 | 1.00 | 1.00 | 4 | 0.78(0.57-0.91) | 0.97(0.95-0.98) |
| DL(Deep Learning) | NA | NA | NA | 5 | 0.76(0.41-0.93) | 0.78(0.40-0.95) |
| NB(Naïve Bayes) | NA | NA | NA | 1 | 0.75 | 0.95 |
| Location of CA Occurrence |  |  |  |  |  |  |
| In-Hospital | 6 | 0.67(0.11-0.97) | 0.49(0.03-0.97) | 5 | 0.58(0.25-0.85) | 0.52(0.18-0.84) |
| Out-of-Hospital | 9 | 0.72(0.37-1.00) | 0.83(0.76-0.91) | 39 | 0.73(0.62-0.82) | 0.81(0.69-0.90) |
| Overall | 15 | 0.72(0.47-0.98) | 0.85(0.79-0.90) | 44 | 0.72(0.61-0.81) | 0.79(0.66-0.88) |
| Scoring system |  |  |  |  |  |  |
| CANPS |  |  |  | 2 | 0.66-0.69 | 1 |
| OCAS |  |  |  | 2 | 0.76-1.00 | 0.69-0.75 |
| TCAHPS |  |  |  | 1 | 0.74 | 0.79 |
| Overall |  |  |  | 5 | 0.30(0.09-0.65) | 0.88(0.12-1.00) |

Note: CANPS: Cardiac Original Article Arrest Neurological Prognosis (CANP) Score, OCAS: Out-of-hospital cardiac arrest score, TCAHPS: The Cardiac Arrest Hospital Prognosis score.

**Figure S1. Forest plot of the C-index meta-analysis of predictive models of favorable neurological outcomes (good cerebral performance category score 1-2) in the training and validation sets.**

**
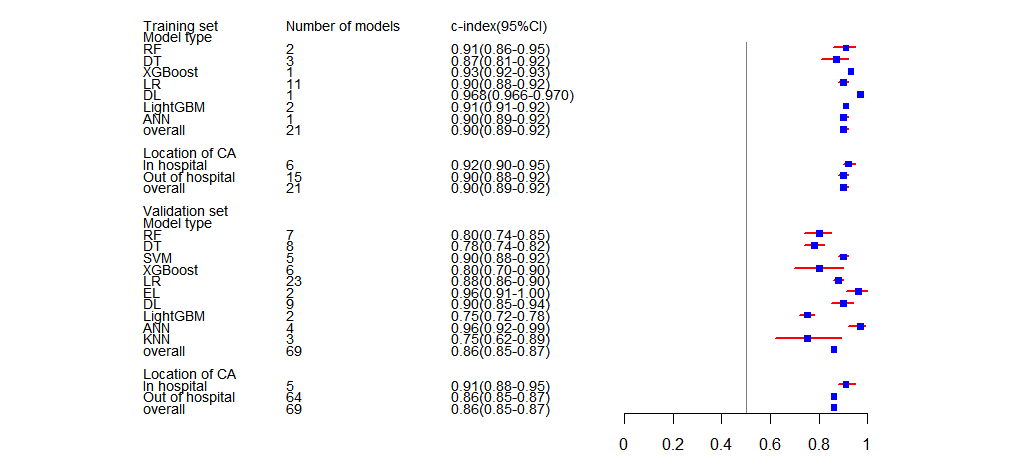
**

**Figure S2. Forest plot of the sensitivity meta-analysis of favorable neurological outcome (good cerebral performance category score 1-2) prediction models in the training and validation sets.**

**
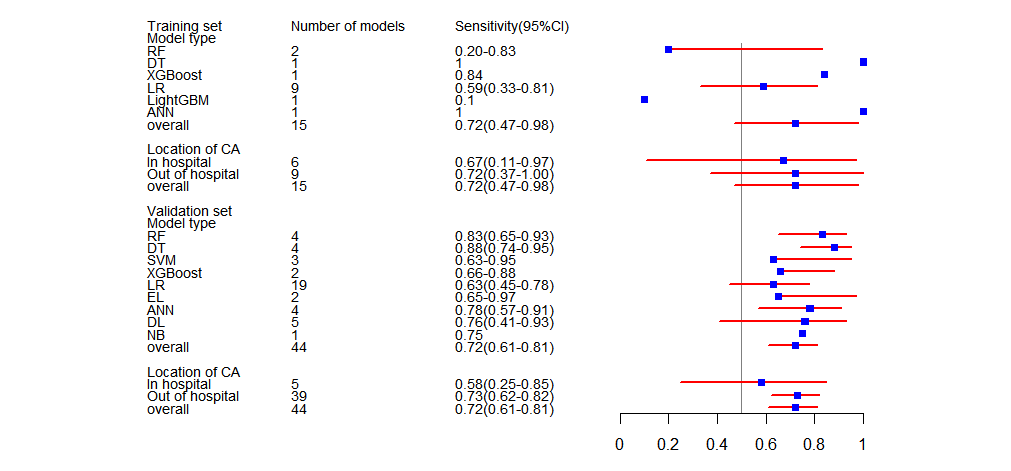
**

**Figure S3. Forest plot of the specificity meta-analysis of prediction models of favorable neurological outcomes (good cerebral performance category score 1-2) in the training and validation sets.**

**
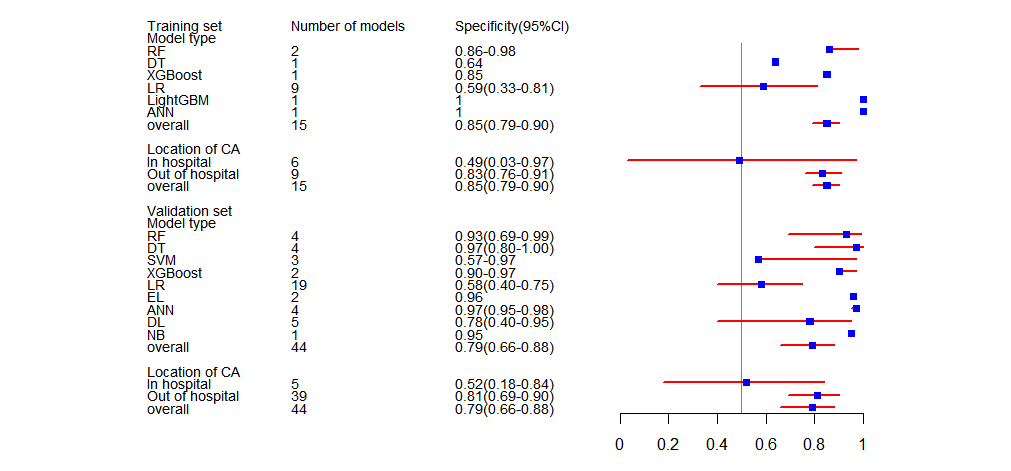
**

**S1. Subgroup analysis for predicting favorable neurological outcomes (good cerebral performance category score 1-2).**

In the data related to in-hospital cardiac arrest, we conducted a meta-subgroup analysis using a random-effects model on the machine learning models predicting CPC1-2 in the training set. The analysis revealed that the C-index, sensitivity, and specificity were 0.92 (95% CI: 0.90-0.95, n=6), 0.67 (95% CI: 0.11-0.97, n=6), and 0.49 (95% CI: 0.03-0.97, n=6), respectively. A similar analysis using a random-effects model on the validation set showed results of 0.91 (95% CI: 0.88-0.95, n=5), 0.58 (95% CI: 0.25-0.85, n=5), and 0.52 (95% CI: 0.18-0.84, n=5), respectively. For out-of-hospital cardiac arrest data, the random-effects model in the training set analysis revealed a C-index, sensitivity, and specificity of 0.90 (95% CI: 0.88-0.92, n=15), 0.73 (95% CI: 0.62-0.82, n=9), and 0.81 (95% CI: 0.69-0.90, n=9), respectively. The validation set analysis showed a C-index, sensitivity, and specificity of 0.86 (95% CI: 0.85-0.87, n=64), 0.73 (95% CI: 0.62-0.82, n=39), and 0.81 (95% CI: 0.69-0.90, n=39), respectively.

**Table S2. Meta-analysis results for the C-index for predicting cardiac arrest mortality.**

| Model type | Training set | | | | Validation set | | | |
| --- | --- | --- | --- | --- | --- | --- | --- | --- |
|  | Events | Sample size | n | C-index(95%CI) | Events | Sample size | n | C-index(95%CI) |
| Machine learning |  |  |  |  |  |  |  |  |
| RF(Random Forest) | 1,986 | 39,602 | 1 | 0.75(0.74-0.76) | 6,662 | 45,859 | 6 | 0.87(0.83-0.91) |
| DT(Decision Tree) | 12,514 | 307,896 | 1 | 0.79(0.79-0.80) | 4,019 | 84,016 | 2 | 0.77(0.69-0.85) |
| SVM(Support Vector Machine) | NA | NA | NA | NA | 2,986 | 15,451 | 3 | 0.84(0.82-0.86) |
| XGBoost | NA | NA | NA | NA | 1,238 | 11,123 | 1 | 0.98(0.97-0.98) |
| LR(Logistic Regression) | 2533 | 5,720 | 7 | 0.83(0.80-0.86) | 3,887 | 20,630 | 11 | 0.83(0.76-0.91) |
| DL(Deep Learning) | NA | NA | NA | NA | 1,979 | 19,924 | 3 | 0.85(0.76-0.95) |
| LightGBM | 13,819 | 197,256 | 2 | 0.86(0.74-0.98) | NA | NA | NA | NA |
| ANN(Artificial Neural Network) | 1,986 | 39,602 | 1 | 0.69(0.67-0.70) | NA | NA | NA | NA |
| HV(Hierarchical vectorizer) | 87,331 | 168,693 | 2 | 0.72(0.70-0.74) | NA | NA | NA | NA |
| MP(Multilayer Perceptron) | NA | NA | NA | NA | 1,923 | 6,381 | 1 | 0.87(0.86-0.88) |
| CR(Cox Regression) | NA | NA | NA | NA | 30 | 53 | 1 | 0.93(0.86-1.00) |
| Location of CA Occurrence |  |  |  |  |  |  |  |  |
| In-Hospital | 88,094 | 170,008 | 4 | 0.84(0.76-0.92) | 664 | 13,083 | 8 | 0.80(0.70-0.90) |
| Out-of-Hospital | 32,075 | 588,761 | 10 | 0.79(0.75-0.83) | 22,060 | 190,354 | 20 | 0.85(0.82-0.89) |
| Overall | 120,169 | 758,769 | 14 | 0.80(0.76-0.84) | 22,724 | 203,437 | 28 | 0.85(0.82-0.87) |
| Scoring system |  |  |  |  |  |  |  |  |
| FACTOR Score |  |  |  |  | 120 | 291 | 2 | 0.83(0.78-0.88) |
| MEWS |  |  |  |  | 111 | 4,559 | 1 | 0.69(0.54-0.85) |
| OCAS |  |  |  |  | 309 | 713 | 1 | 0.81(0.78-0.84) |
| SAPSⅡ |  |  |  |  | 21 | 111 | 1 | 0.71(0.62-0.80) |
| TCAHPS |  |  |  |  | 309 | 713 | 1 | 0.83(0.80-0.86) |
| Overall |  |  |  |  | 870 | 6,387 | 6 | 0.81(0.78-0.84) |

Note: MEWS: Modified early warning score, OCAS: Out-of-hospital cardiac arrest score, SAPSⅡ: Simplified Acute Physiology Score II, TCAHPS: The Cardiac Arrest Hospital Prognosis score.

**Table S3. Meta-analysis results for the sensitivity and specificity of mortality rate prediction models for cardiac arrest.**

| Model type | Training set | | | Validation set | | |
| --- | --- | --- | --- | --- | --- | --- |
|  | n | Sensitivity(95%CI) | Specificity(95%CI) | n | Sensitivity(95%CI) | Specificity(95%CI) |
| Machine learning |  |  |  |  |  |  |
| RF(Random Forest) | NA | NA | NA | 5 | 0.84(0.74-0.91) | 0.78(0.67-0.86) |
| DT(Decision Tree) | NA | NA | NA | 1 | 0.68 | 0.68 |
| SVM(Support Vector Machine) | NA | NA | NA | 3 | 0.68-0.92 | 0.66-0.78 |
| XGBoost | NA | NA | NA | 1 | 0.92 | 0.93 |
| LR(Logistic Regression) | 5 | 0.67(0.44-0.84) | 0.55(0.28-0.79) | 9 | 0.84(0.78-0.88) | 0.74(0.67-0.80) |
| DL(Deep Learning) | NA | NA | NA | 2 | 0.87-0.91 | 0.48-0.78 |
| LightGBM | 1 | 0.99 | 0.12 | NA | NA | NA |
| HV(Hierarchical vectorizer) | 1 | 0.35 | 0.97 | NA | NA | NA |
| MP(Multilayer Perceptron) | NA | NA | NA | 1 | 0.9 | 0.71 |
| CR(Cox Regression) | NA | NA | NA | 1 | 0.96 | 0.78 |
| Location of CA Occurrence |  |  |  |  |  |  |
| In-Hospital | 3 | 0.35-0.85 | 0.68-0.97 | 5 | 0.78(0.66-0.87) | 0.78(0.74-0.82) |
| Out-of-Hospital | 4 | 0.80(0.61-0.99) | 0.63(0.16-1.00) | 18 | 0.84(0.80-0.88) | 0.79(0.74-0.84) |
| Overall | 7 | 0.82(0.58-0.94) | 0.76(0.51-0.91) | 23 | 0.83(0.79-0.87) | 0.79(0.74-0.83) |
| Scoring system |  |  |  |  |  |  |
| FACTOR score |  |  |  | 2 | 0.72-0.74 | 0.71-0.83 |
| MEWS |  |  |  | 1 | 0.75 | 0.57 |
| OCAS |  |  |  | 1 | 0.77 | 0.7 |
| SAPSⅡ |  |  |  | 1 | 0.54 | 0.84 |
| TCAHPS |  |  |  | 1 | 0.76 | 0.77 |
| Overall |  |  |  | 6 | 0.75(0.71-0.78) | 0.74(0.66-0.81) |

Note: MEWS: Modified early warning score, OCAS: Out-of-hospital cardiac arrest score, SAPSⅡ: Simplified Acute Physiology Score II, TCAHPS: The Cardiac Arrest Hospital Prognosis score.

**Figure S4. Forest plot of the C-index meta-analysis of prediction models of cardiac arrest mortality rate in the training and validation sets.**


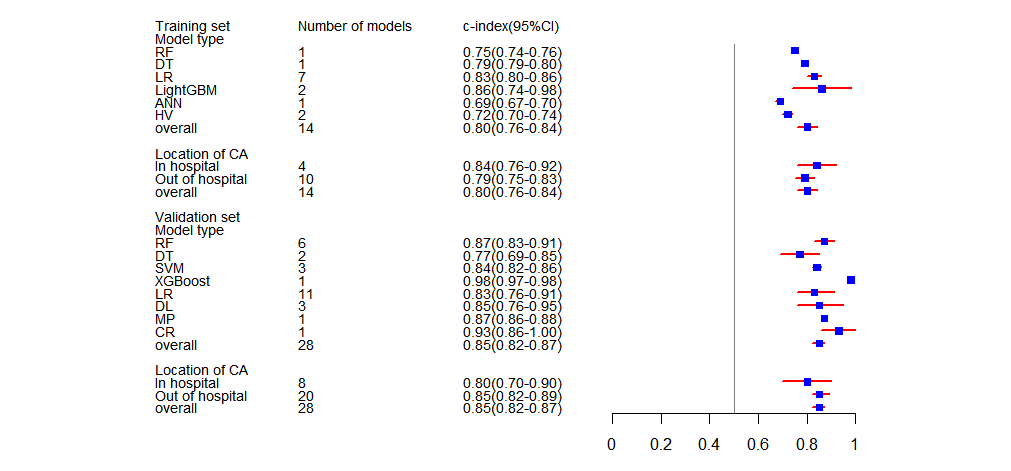


**Figure S5. Forest plot of the sensitivity meta-analysis of cardiac arrest mortality rate prediction models in the training and validation sets.**

**
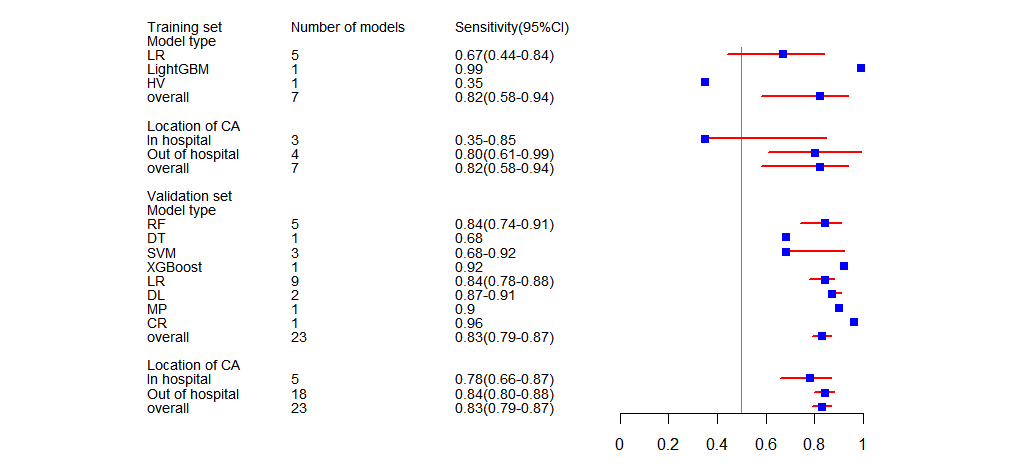
**

**Figure S6. Forest plot of the specificity meta-analysis of cardiac arrest mortality rate prediction models in the training and validation sets.**

**
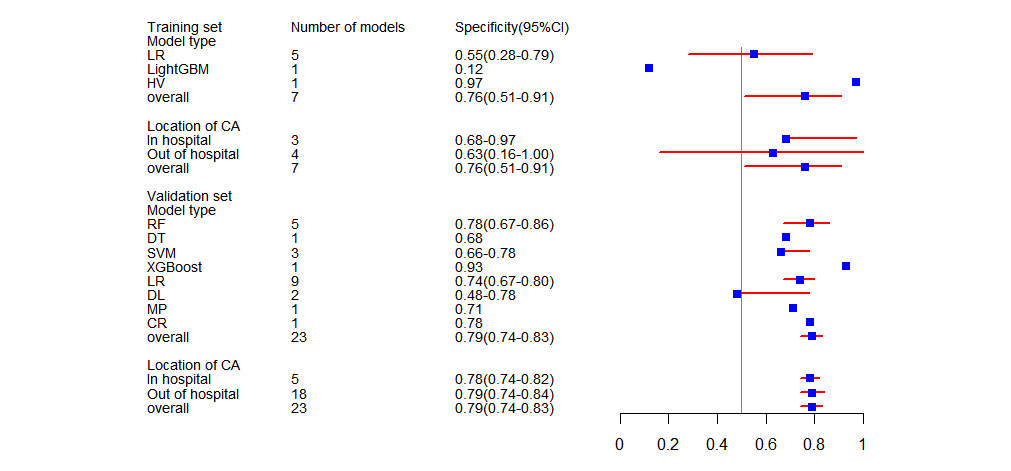
**

**S2. Subgroup analysis for predicting cardiac arrest mortality.**

In the in-hospital cardiac arrest data, a meta-subgroup analysis using a random-effects model was performed on the machine learning models predicting CAM in the training set. The analysis revealed a C-index, sensitivity, and specificity of 0.84 (95% CI: 0.76-0.92, n=4), 0.35-0.85, and 0.68-0.97, respectively. The validation set analysis showed a C-index, sensitivity, and specificity of 0.80 (95% CI: 0.70-0.90, n=8), 0.77 (95% CI: 0.67-0.85, n=5), and 0.79 (95% CI: 0.74-0.83, n=5), respectively. For out-of-hospital cardiac arrest data, the random-effects model in the training set analysis revealed a C-index, sensitivity, and specificity of 0.79 (95% CI: 0.75-0.83, n=10), 0.80 (95% CI: 0.61-0.99, n=4), and 0.63 (95% CI: 0.16-1.00, n=4), respectively. The validation set analysis showed a C-index, sensitivity, and specificity of 0.85 (95% CI: 0.82-0.89, n=20), 0.84 (95% CI: 0.80-0.88, n=18), and 0.79 (95% CI: 0.74-0.84, n=18), respectively.

**Table S4. Meta-analysis results for the C-index for return of spontaneous circulation prediction models.**

| Model type | Training set | | | | Validation set | | | |
| --- | --- | --- | --- | --- | --- | --- | --- | --- |
|  | Events | Sample size | n | C-index(95%CI) | Events | Sample size | n | C-index(95%CI) |
| Machine learning |  |  |  |  |  |  |  |  |
| RF(Random Forest) | 3099 | 7,785 | 3 | 0.83(0.75-0.92) | 5,126 | 54,684 | 4 | 0.78(0.71-0.84) |
| DT(Decision Tree) | NA | NA | NA | NA | NA | NA | 1 | 0.808(0.807-0.810) |
| SVM(Support Vector Machine) | NA | NA | NA | NA | NA | NA | 2 | 0.79(0.66-0.92) |
| LR(Logistic Regression) | 3,751 | 11,359 | 5 | 0.85(0.71-0.98) | 3187 | 14,209 | 3 | 0.74(0.67-0.82) |
| DL(Deep Learning) | NA | NA | NA | NA | NA | NA | 1 | 0.828(0.826-0.830) |
| LightGBM | 11,996 | 157,654 | 1 | 0.86(0.86-0.87) | NA | NA | NA | NA |
| ANN(Artificial Neural Network) | 2,319 | 6,525 | 1 | 0.72(0.71-0.73) | 468 | 1632 | 1 | 0.712(0.711-0.713) |
| KNN(K-Nearest Neighbor) | NA | NA | NA | NA | NA | NA | 1 | 0.785(0.782-0.788) |
| Location of CA Occurrence |  |  |  |  |  |  |  |  |
| In-Hospital | 1,908 | 2,962 | 4 | 0.90(0.897-0.904) | NA | NA | NA | NA |
| Out-of-Hospital | 19,257 | 180,361 | 6 | 0.79(0.70-0.87) | 8781 | 70,525 | 13 | 0.77(0.74-0.80) |
| Overall | 21,165 | 183,323 | 10 | 0.83(0.79-0.88) | 8781 | 70,525 | 13 | 0.77(0.74-0.80) |
| Scoring system |  |  |  |  |  |  |  |  |
| The ROSC after cardiac arrest score | NA | NA | NA | NA | 1557 | 18,918 | 1 | 0.73(0.72-0.74) |
| Overall | NA | NA | NA | NA | 1557 | 18,918 | 1 | 0.73(0.72-0.74) |

**Table S5. Meta-analysis results for the sensitivity and specificity of return of spontaneous circulation prediction models.**

| Model type | Training set | | | Validation set | | |
| --- | --- | --- | --- | --- | --- | --- |
|  | n | Sensitivity(95%CI) | Specificity(95%CI) | n | Sensitivity(95%CI) | Specificity(95%CI) |
| Machine learning |  |  |  |  |  |  |
| RF(Random Forest) | 2 | 0.30-0.84 | 0.69-0.96 | 3 | 0.28-0.85 | 0.60-0.97 |
| LR(Logistic Regression) | 4 | 0.68(0.33-1.00) | 0.87(0.82-0.82) | 2 | 0.27-0.77 | 0.63-0.97 |
| LightGBM | 1 | 0.04 | 1.00 | NA | NA | NA |
| ANN(Artificial Neural Network) | 1 | 0.30 | 0.97 | 1 | 0.28 | 0.97 |
| Location of CA Occurrence |  |  |  |  |  |  |
| In-Hospital | 2 | 0.83-0.84 | 0.69-0.85 | NA | NA | NA |
| Out-of-Hospital | 6 | 0.42(0.24-0.59) | 0.94(0.91-0.96) | 6 | 0.53(0.31-0.74) | 0.88(0.71-0.96) |
| Overall | 8 | 0.52(0.31-0.73) | 0.91(0.88-0.93) | 6 | 0.53(0.31-0.74) | 0.88(0.71-0.96) |
| Scoring system |  |  |  |  |  |  |
| The ROSC after cardiac arrest score |  |  |  | 1 | 0.72 | 0.62 |
| Overall |  |  |  | 1 | 0.72 | 0.62 |

**Figure S7. Forest plot of the C-index meta-analysis of return of spontaneous circulation prediction models in the training and validation sets.**


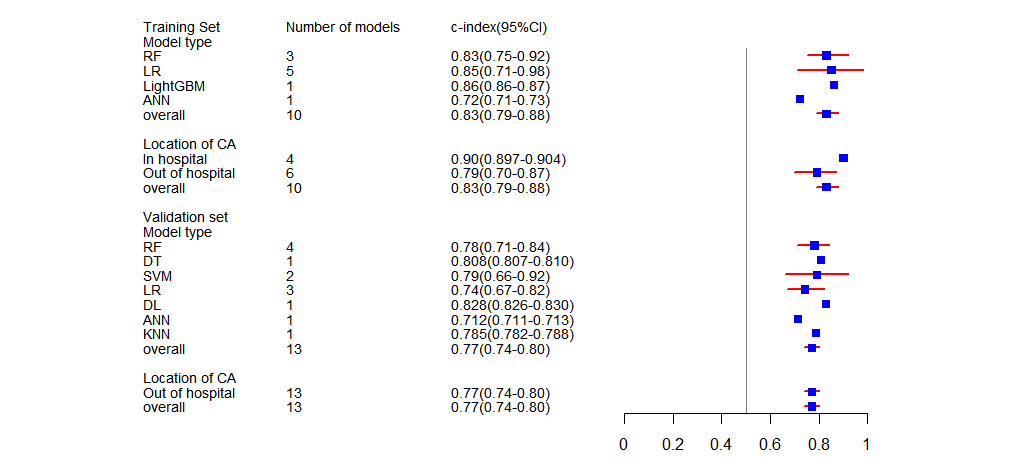


**Figure S8. Forest plot of the sensitivity meta-analysis of return of spontaneous circulation prediction models in the training and validation sets.**


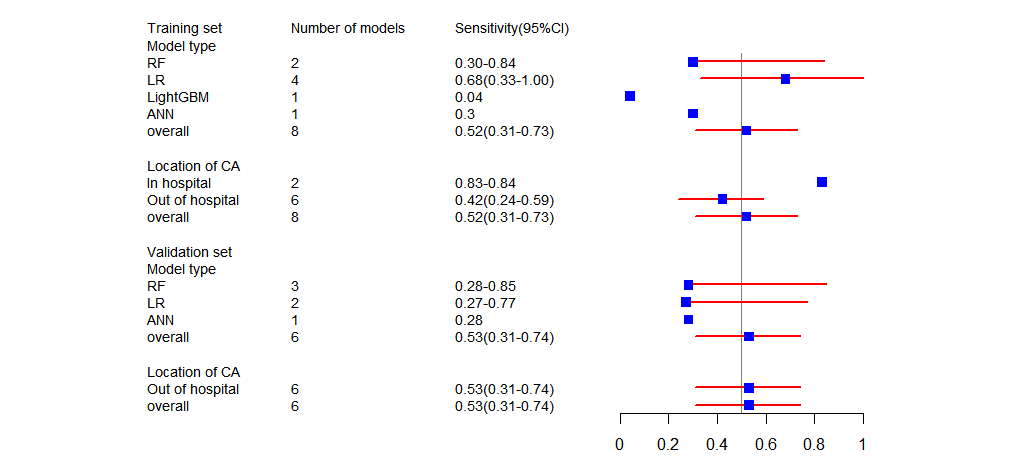


**Figure S9. Forest plot of the specificity meta-analysis of return of spontaneous circulation prediction models in the training and validation sets.**


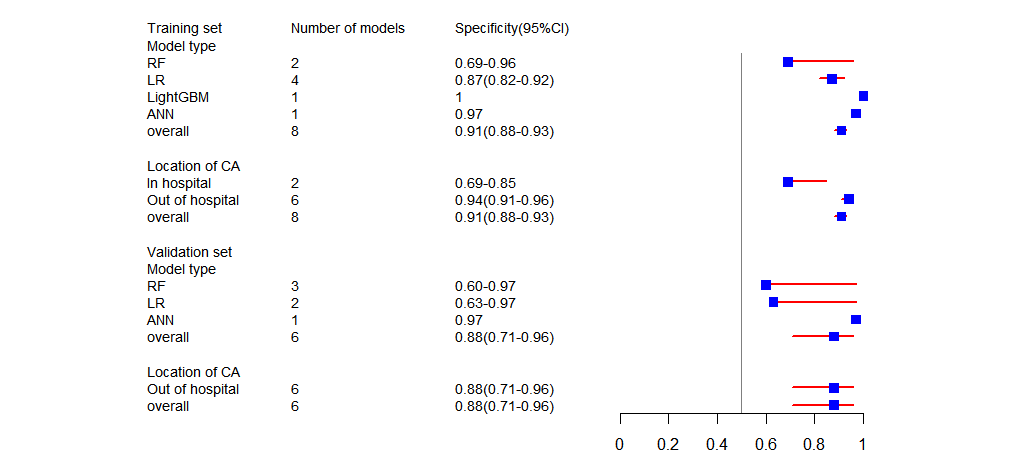


**S3. Subgroup analysis for predicting return of spontaneous circulation.**

In the in-hospital cardiac arrest data, a meta-subgroup analysis using a random-effects model was performed on the machine learning models predicting ROSC in the training set. The analysis showed that the C-index, sensitivity, and specificity were 0.90 (95% CI: 0.897-0.904, n=4), 0.83-0.84, and 0.69-0.85, respectively. Since the original studies did not provide validation set data, we did not perform a meta-subgroup analysis for the validation set of this outcome. In the out-of-hospital cardiac arrest data, the random-effects model in the training set analysis revealed a C-index, sensitivity, and specificity of 0.79 (95% CI: 0.70-0.87, n=6), 0.42 (95% CI: 0.24-0.59, n=6), and 0.94 (95% CI: 0.91-0.96, n=6), respectively. The validation set analysis showed a C-index, sensitivity, and specificity of 0.77 (95% CI: 0.74-0.80, n=13), 0.53 (95% CI: 0.31-0.74, n=6), and 0.88 (95% CI: 0.71-0.96, n=6), respectively.

**Table S6. Analysis results of variable weights for predicting cardiac arrest and favorable neurological outcomes (good cerebral performance category score 1-2) using machine learning models.**

| **Cardiac Arrest**（**N=28**） | | **The good-cerebral performance category score(CPC 1-2)**（IHCA, **N=10; OHCA, N=32**） | | | |
| --- | --- | --- | --- | --- | --- |
| **Variable** | **n** | **Variable** | **IHCA, n** | **Variable** | **OHCA, n** |
| RR | 22 | Rhythm(shockable/non-shockable) | 8 | Age | 25 |
| BP | 20 | Age | 7 | Rhythm(shockable/non-shockable) | 24 |
| Age | 19 | Medication use | 6 | Medication use | 18 |
| Temperature | 19 | Gender | 5 | ROSC | 14 |
| Oxygen Saturation | 15 | GCS | 5 | Gender | 12 |
| Airway | 9 |  |  | no-flow time(resuscitation duration) | 12 |
|  |  |  |  | EMS transport（scene interval,arrival time,response time) | 12 |
|  |  |  |  | Defibrillation | 11 |
|  |  |  |  | GCS | 6 |

Note: IHCA: In-Hospital Cardiac Arrest, OHCA: Out-of-Hospital Cardiac arrest, RR: Respiratory rate, BP: Blood pressure, ROSC: Return of spontaneous circulation, GCS: Glasgow Coma Scale.

**Table S7. Machine learning model variables.**

| **Modeling variables** | | **Outcome, N** | | |
| --- | --- | --- | --- | --- |
|  |  | **Cardiac Arrest (N=28)** | **The good-cerebral performance category score(CPC 1-2)** | |
|  |  | N=28 | IHCA, N=10 | OHCA, N=32 |
| **Demographic characteristics** | Age | 19 | 7 | 25 |
|  | Gender | 10 | 5 | 12 |
|  | Height | 2 | 0 | 1 |
|  | Weight | 2 | 0 | 1 |
|  | BMI | 1 | 0 | 0 |
|  | Race | 0 | 0 | 1 |
| **Vital signs** | Pulse | 10 | 0 | 0 |
|  | HR | 12 | 1 | 0 |
|  | RR | 22 | 0 | 0 |
|  | Temperature | 19 | 1 | 0 |
|  | BP | 20 | 0 | 3 |
|  | Oxygen Saturations | 15 | 0 | 0 |
|  | Blood gas analysis results（FiO2、PaO2、PCO2、OI） | 2 | 2 | 0 |
|  | Pain | 3 | 0 | 0 |
|  | Pupillary reflex | 0 | 0 | 4 |
|  | Consciousness | 11 | 0 | 0 |
| **Scale** | GCS | 5 | 5 | 6 |
|  | MEWS | 1 | 0 | 0 |
|  | APACHE II Score | 1 | 1 | 0 |
|  | SOFA | 0 | 1 | 0 |
| **Imaging** | Brain CT | 0 | 0 | 4 |
|  | Coronary Angiography | 0 | 0 | 1 |
|  | Diffusion-weighted imaging | 0 | 0 | 1 |
|  | C-reactive protein | 1 | 0 | 0 |
|  | Blood Cells Counts (Platelets, Neutrophils, Hemoglobin, Whigt Blood Cell) | 3 | 1 | 3 |
| **Cardiac function index & coagulation index** | Brain natriuretic peptide | 1 | 0 | 0 |
|  | Prothrombin activity | 1 | 0 | 0 |
|  | Prothrombin time | 1 | 0 | 0 |
|  | Activated Partial Thromboplastin Time | 1 | 1 | 0 |
|  | Cardiac troponin I | 1 | 0 | 1 |
| **Biochemical indexes** | Total Bilirubin | 2 | 0 | 1 |
|  | ALT | 1 | 0 | 0 |
|  | Glucose | 3 | 0 | 3 |
|  | Albumin | 2 | 1 | 0 |
|  | pH | 1 | 3 | 6 |
|  | Electrolyte level (Sodium, Potassium, HCO3-) | 2 | 2 | 7 |
|  | Base deficit | 0 | 1 | 2 |
|  | Lactate | 0 | 3 | 6 |
|  | Creatinine | 2 | 4 | 3 |
|  | Blood urea nitrogen | 1 | 0 | 1 |
|  | Heart Rate Variability(HRV) | 4 | 0 | 0 |
|  | Do-Not-Resuscitate | 1 | 0 | 0 |
|  | Triage | 2 | 0 | 0 |
|  | Etiology | 0 | 2 | 4 |
|  | Symptoms/chief concern | 3 | 0 | 0 |
|  | Diagnosis | 1 | 1 | 0 |
|  | Comorbidities | 1 | 0 | 1 |
| **Medical history/Disease** | Myocardial infarction(Killip class,segment | 3 | 0 | 2 |
|  | Arrhythmia | 1 | 0 | 2 |
|  | Pulmonary disease | 3 | 1 | 4 |
|  | Heart disease | 2 | 0 | 2 |
|  | Shock | 2 | 1 | 2 |
|  | Renal disease(chronic kidney disease | 3 | 0 | 1 |
|  | Nervous system disease(dementia,syncope,seizure | 2 | 0 | 1 |
|  | Artery disease(hypertension | 0 | 0 | 3 |
|  | Digestive system disease | 0 | 0 | 1 |
|  | Electrolyte disturbance | 0 | 0 | 3 |
|  | Hyperlipidemia | 1 | 0 | 1 |
|  | Diabetes | 2 | 0 | 5 |
|  | Obesity | 1 | 0 | 0 |
|  | Malignancy | 1 | 0 | 2 |
|  | Past surgical history | 1 | 0 | 0 |
| **Time/duration** | Recorded time of each vital sign | 2 | 1 | 0 |
|  | Discharge time | 1 | 0 | 0 |
|  | Duration of ECG | 1 | 0 | 0 |
|  | Collapse/arrest duration | 1 | 1 | 6 |
|  | No-flow time(resuscitation duration) | 0 | 1 | 12 |
|  | Low-flow time | 0 | 2 | 4 |
| **EMS** | EMS transport (scene interval, arrival time, response time), setting | 4 | 3 | 12 |
|  | Witnessed (location, status) | 2 | 1 | 10 |
|  | ED occupancy | 1 | 0 | 0 |
| **BLS&ACLS** | CPR（duration) | 1 | 3 | 5 |
|  | Airway support(oxygen supplement) | 9 | 1 | 6 |
|  | Defibrillation | 1 | 0 | 11 |
|  | rhythm(shockable/non-shckable) | 1 | 8 | 24 |
|  | ROSC/NROSC | 0 | 1 | 14 |
|  | ECMO | 0 | 0 | 1 |
|  | Vessel(access,coronary revascularisation,intravenous line | 0 | 0 | 3 |
|  | Medication use (Epinephrine, Norepinephrine, Dopamine, Antiplatelet, Anticoagulant, Benzodiazepine, Vasopressor) | 2 | 6 | 18 |
